# Thromboxane signalling links immune activation to enhanced glucose uptake in skeletal muscle

**DOI:** 10.1007/s00125-026-06684-8

**Published:** 2026-02-20

**Authors:** Ahmed M. Abdelmoez, Maxence Jollet, Xue Yu, David Rizo-Roca, Alesandra A. Marica, Joaquin Ortiz de Zevallos, Lucile Dollet, Melissa L. Borg, Marie Björnholm, Antonio Checa, Tommy Olsson, Julia Otten, Juleen R. Zierath, Anna Krook, Thue W. Schwartz, Alexander V. Chibalin, Nicolas J. Pillon

**Affiliations:** 1 Department of Physiology and Pharmacology, Karolinska Institutet, Stockholm, Sweden; 2 Department of Molecular Medicine and Surgery, Karolinska Institutet, Stockholm, Sweden; 3 Unit of Integrative Metabolomics, Institute of Environmental Medicine, Karolinska Institutet, Stockholm, Sweden; 4 Department of Public Health and Clinical Medicine, Umeå University, Umeå, Sweden; 5 Novo Nordisk Foundation Center for Basic Metabolic Research, University of Copenhagen, Copenhagen, Denmark

**Keywords:** Exercise, Glucose, Metabolism, Skeletal muscle, Thromboxane, Type 2 diabetes

## Abstract

**Aims/hypothesis:**

Exercise elicits a spectrum of metabolic and inflammatory responses that are crucial for skeletal muscle adaptation and overall health, particularly in the context of metabolic diseases, yet the contribution of prostanoid signalling to these processes remains unclear. We hypothesised that exercise-induced thromboxane production enhances skeletal muscle glucose uptake and improves whole-body glucose control.

**Methods:**

Plasma prostanoids were quantified in men and women with normal glucose tolerance or type 2 diabetes before, immediately after and 3 h after a single bout of exercise. Cyclooxygenase (COX-2) transcript levels were evaluated in human skeletal muscle, whole blood, peripheral blood mononuclear cells and skeletal muscle-resident immune cells. Metabolic and transcriptomic effects of thromboxane receptor activation were analysed in mouse C2C12, rat L6 and human primary skeletal muscle cells. Glucose tolerance in vivo was assessed following i.p. administration of the thromboxane receptor agonist I-BOP in male and female mice. Tissue-specific glucose uptake was quantified by measuring radiolabelled 2-deoxyglucose incorporation during an IVGTT.

**Results:**

Acute exercise increased plasma thromboxane B₂ concentrations and skeletal muscle mRNA levels of *PTGS2* (encoding COX-2) selectively in monocyte/macrophage populations. In skeletal muscle cells, the thromboxane receptor agonist I-BOP increased glucose uptake in a dose-dependent manner up to 2.5-fold within 4 h and enhanced glycogen synthesis by 430%. Transcriptomic and signalling analysis revealed activation of protein kinase A and cytoskeletal remodelling pathways linked to GLUT4 trafficking. In vivo, I-BOP improved glucose tolerance in male mice in a dose-dependent manner, without altering insulin levels. Thromboxane receptor stimulation increased glucose uptake in extensor digitorum longus muscle by 43%. Importantly, thromboxane receptor activation preserved its glucose-lowering efficacy in diet-induced obese male mice.

**Conclusions/interpretation:**

Exercise induces skeletal muscle-derived thromboxane production through macrophage-specific COX-2 activation. Thromboxane receptor stimulation enhances glucose uptake and glycogen storage via cytoskeletal remodelling, partially mimicking the acute exercise transcriptomic response. In vivo, thromboxane receptor activation improves glucose tolerance and skeletal muscle glucose uptake, with preserved efficacy in obesity. These findings identify thromboxane signalling as a previously unrecognised immunometabolic axis linking inflammation to glucose regulation and highlight the thromboxane receptor as a potential therapeutic target for metabolic disease.

**Graphical Abstract:**

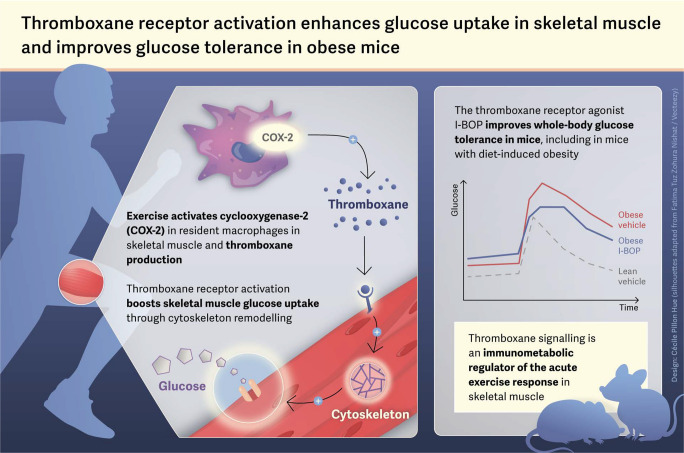

**Supplementary Information:**

The online version of this article (10.1007/s00125-026-06684-8) contains peer-reviewed but unedited supplementary material.


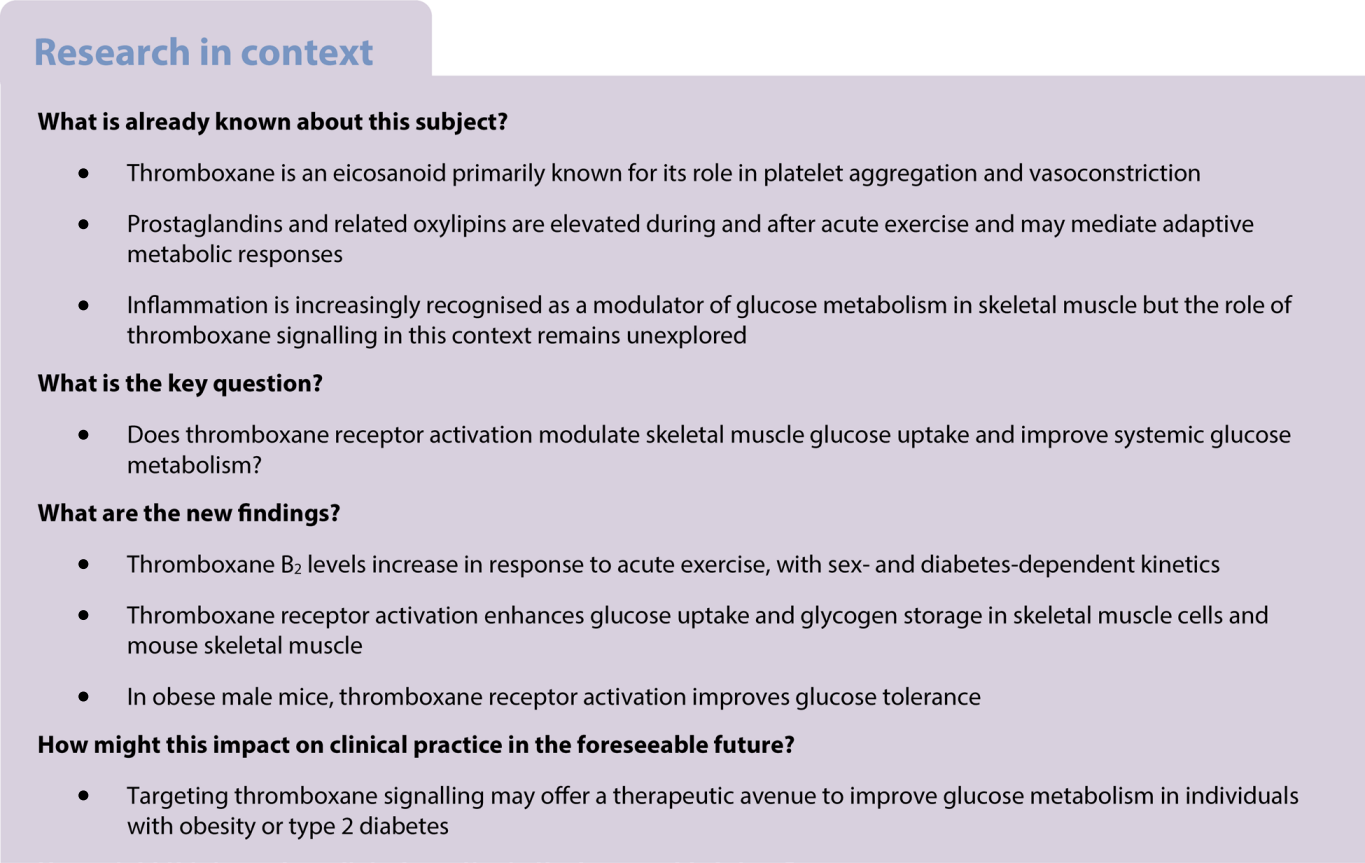


## Introduction

Inflammatory responses are increasingly recognised as essential mediators in the pathogenesis of cardiometabolic diseases [1]. Immune cell infiltration into adipose tissue, liver and skeletal muscle is associated with complications in obesity and type 2 diabetes [2]. While inflammatory responses can be detrimental, they simultaneously play a beneficial reparative function during tissue remodelling, such as post injury or following acute exercise [3]. However, immune response dysregulation can impede recovery, promoting fibrosis and compromising tissue regeneration [3]. Consequently, maintaining a finely regulated immune response is crucial for metabolic homeostasis and the ability of an organism to adapt to environmental stressors [4–6].

Inflammatory responses comprise a complex communication network of soluble molecules coordinating immune cell interactions. Cytokines and chemokines, such as IL-6 and CCL2, are released by skeletal muscle cells in response to metabolic stressors such as nutrient overload or muscle contraction [7]. Additionally, other small molecules, including lactate, succinate and ATP, are secreted by skeletal muscle to recruit and activate immune cells [8, 9]. Among these molecules, oxylipins, which are bioactive lipid mediators, are rapidly synthesised in response to infection and injury. Oxylipins are a large class of molecules derived from polyunsaturated fatty acids and include eicosanoids, which encompass prostaglandins, leukotrienes and thromboxane. Oxylipins exhibit multifaceted immunomodulatory functions, from vasodilation to coagulation and allergic response regulation [10]. Prostaglandins exemplify this complexity, being capable of both promoting and resolving inflammation, while modulating the production of cytokines, immune cell recruitment and vascular permeability [11]. Thromboxane, primarily known for a role in platelet aggregation and vasoconstriction, also contributes to immune responses by modulating the activity of leukocytes [12]. Leukotrienes, produced by lipoxygenase pathways, are potent chemoattractants that drive the migration of neutrophils and other immune cells to sites of tissue damage or infection [13]. Collectively, these molecular mediators underscore the intricate and dynamic nature of inflammatory signalling, highlighting the precision required for effective immune responses.

Acute exercise increases the circulatory levels of immune cells and activates inflammatory pathways, both systemically and within skeletal muscle [6, 14, 15]. Epidemiological and molecular investigations have consistently demonstrated elevated oxylipin concentrations in plasma and skeletal muscle during and following acute exercise, suggesting a coordinated molecular response to physical exertion [16–20]. In addition, acute aerobic exercise induces thromboxane receptor phosphorylation in skeletal muscle, providing evidence for eicosanoid pathway modification [21]. Despite these insights, current understanding of the role of thromboxane in exercise responses has been largely derived from studies in young, healthy, male volunteers, thereby presenting a knowledge gap regarding regulatory mechanisms in individuals with metabolic diseases.

Despite the proinflammatory effects of acute exercise, regular physical activity provides substantial metabolic benefits, including enhanced skeletal muscle glucose uptake and improved systemic glycaemic control [22]. While inflammatory responses potentially mediate these beneficial adaptations, the soluble mediators of exercise-induced acute inflammation and chronic metabolic inflammation remain understudied. Here, we investigate the mechanistic role of exercise-induced oxylipins in modulating metabolic responses in type 2 diabetes, characterising oxylipin profiles through studies of human plasma samples, cell cultures and animal models. Our study examines how acute exercise-triggered thromboxane production differentially mediates metabolic responses, focusing on thromboxane receptor activation and the impact on glucose metabolism.

## Methods

### Clinical studies

The study was conducted in accordance with the Declaration of Helsinki, with all participants providing informed consent. The ethics committees in Stockholm and at Umeå University approved the study protocols. The study was performed in Stockholm and Umeå, Sweden. Participants were recruited through local advertising. The cohort comprised sedentary men and women with normal glucose tolerance or type 2 diabetes. Sex was self-identified and obtained from medical records. Information on gender identity, race or ethnicity was not collected; therefore, the representativeness of the cohort relative to the general Swedish population cannot be assessed. Clinical characteristics of the study participants are presented in electronic supplementary material (ESM) Table 1. Exclusion criteria were BMI >33 kg/m^2^, physical impairment, CVD, smoking status and treatment with insulin, β-blockers or anti-inflammatory agents. Participants underwent an OGTT to verify normal glucose tolerance. Body composition was determined using dual x-ray absorptiometry. On the experimental day, participants arrived at the laboratory after a light breakfast and completed an acute exercise session on a cycle ergometer (Rodby). The exercise intensity was maintained at 85% of the individual maximal heart rate for 30 min. Blood samples were collected before exercise, immediately post-exercise and after a 3 h rest period and stored for subsequent analysis. Clinical chemistry was performed at Karolinska University Hospital. EDTA-plasma was used for oxylipin analysis.

### Animal experiments

All experimental procedures were approved by the Stockholm North Animal Ethics Committee. Male and female C57BL/6J mice were obtained from Charles River Laboratories and housed in groups under a 12 h light–dark cycle with ad libitum access to water and standard rodent chow.

### Acute exercise in male and female mice

Five-month-old male and female C57BL/6J mice performed an acute high-intensity interval training (HiiT) session on a treadmill. Basal blood samples were collected from the tail vein before exercise and immediately after exercise using heparinised glass capillaries. Details of the exercise protocol are described in ESM Methods.

### Oxylipin analysis

Plasma collected after exercise in human and mice was extracted as described [23] and oxylipins were measured by LC–tandem MS (LC-MS/MS). Details of the extraction method are given in ESM Methods. Standards used for quantification, retention time and selected reaction monitoring transition for each oxylipin are detailed in ESM Table 2. Plasma oxylipin concentrations were log_2_-transformed and analysed using principal component analysis (PCA) to assess batch effects. Outlier identification was performed using a threshold of ±10 SDs from the mean. Compounds with over 20% missing data were excluded and remaining missing values were imputed using the K-nearest neighbours (KNN) method, using K=10, optimised to the smallest sample group. Statistical analysis of oxylipin levels was conducted using a multi-level linear approach (limma), with participant pairing adjustments (ESM dataset). The design matrix incorporated main effects of disease status, sex and exercise timepoints (pre, post, rest), along with interactions. Statistical significance was determined using the Benjamini–Hochberg method to control for false discovery rates (FDRs) across multiple comparisons.

### Transcriptional analysis of PTGS2 in response to exercise across human and mouse tissues

Public transcriptomic datasets were retrieved from the Gene Expression Omnibus (GEO) repository (https://www.ncbi.nlm.nih.gov/geo/) to evaluate the expression of PTGS2 (encoding cyclooxygenase-2, COX-2) in response to exercise across human and mouse tissues. Datasets were selected based on the availability of samples collected ≤6 hours post-exercise to focus on acute transcriptional responses. Only datasets with available metadata indicating timepoint, tissue, and experimental condition were included. All selected datasets are listed in ESM Table 3. Raw data were downloaded and re-analysed using standardised pipelines. For RNA-seq datasets, raw counts or transcripts per million (TPMs) were imported, low-expressed genes filtered, and normalised using the trimmed mean of M-values (TMM) method followed by log₂-transformation. Microarray datasets were log₂-transformed, expression-filtered, and annotated using probe-to-gene mappings. For each dataset, PTGS2 expression was extracted and normalised to pre-exercise (baseline) samples to control for inter-study variability. Metadata were manually curated from GEO series matrix files and supplementary files. Harmonised variables included GEO accession, species, tissue type, subject ID, sex, diagnosis status, protocol, and timepoint. All analyses were performed in R (v4.3.0). Gene annotations were retrieved from the org.Hs.eg.db and Homo.sapiens Bioconductor packages.

### IPGTT in chow- or high-fat-diet-fed mice

Experiments were performed using 16- to 20-week-old male and female mice. For the study of obesity, 6-week-old male mice were fed a high-fat diet (60% energy from fat; D12492, Research Diet, New Brunswick, NJ) for 12 weeks, with experiments conducted when mice were 18 weeks old. After 3 h fasting, mice were randomised to receive an i.p. injection of the thromboxane receptor agonist I-BOP (Sigma Aldrich, SML0504; 5–100 µg/kg) or vehicle (PBS). The experimenter performing the injections was aware of group assignments but all other experimenters involved in data collection and outcome assessment were blinded to treatment.

One hour later, glucose (1.5 g/kg) was administered by i.p. injection. Blood glucose and insulin levels were measured using a glucose meter (OneTouch Ultra Glucose Meter; LifeScan) and ELISA kit (Ultra-sensitive Mouse Insulin ELISA Kit; Crystal Chem, Downers Grove, IL), respectively. Blood was sampled from the tail vein.

### Tissue-specific glucose uptake assay using 2-deoxy[^14^C]glucose

Seven days prior to the analysis, 20-week-old mice were anaesthetised using constant isoflurane (2.6%) and implanted with a jugular vein catheter (C20PU-MJV20; Instech Technologies) attached to a transcutaneous vascular access button (VABM1B; Instech Technologies). On the day of the analysis, the mice were fasted for 4 h. I-BOP was prepared by evaporating the solvent and resuspending the compound in DPBS. Then, all mice were randomised to receive an i.v. administration of a mixture containing 2 g/kg glucose (HP Halden Pharma, Halden Norway), 0.11 MBq of 2-deoxy-d-[1-^14^C]glucose (PerkinElmer, Waltham, MA) and 20 µg/kg of the thromboxane receptor agonist I-BOP (Sigma Aldrich, SML0504) or vehicle (PBS) in a total volume of 150 µl. Blood was sampled from the tail vein using heparinised capillary tubes at 0, 3, 6, 10, 15, 30, 40 and 60 min and immediately mixed with ZnSO_4_ (0.3 mol/l) and BaOH_2_ (0.3 mol/l) before centrifugation (13,000 g, 2 min) for the determination of 2-deoxy-d-[1-^14^C]glucose concentration. Mice were euthanised at the end of the experiment (60 min) by an overdose of pentobarbital, and tissues were harvested. The tissue content of 2-deoxy-d-[1-^14^C]glucose and 2-deoxy-d-[1-^14^C]glucose 6-phosphate was then determined as described previously [24]. Individual tissue glucose uptake was quantified by calculating the intracellular accumulation of radiolabelled 2-deoxyglucose as the difference between total 2-deoxyglucose and phosphorylated 2-deoxyglucose, normalised to tissue protein content determined by the Bradford assay. Three samples with very low counts (less than 50 cpm/mg protein) were excluded from the analysis. To account for differences in tracer delivery between animals, tissue cpm were further normalised to the AUC of blood cpm over time, yielding the rate of glucose uptake (Rg).

### Measurement of plasma variables

Plasma concentrations of NEFA, triacylglycerol (TAG), ketone bodies and glycerol were quantified using colorimetric enzymatic assays. NEFA levels were measured using the NEFA-HR assay kit (FUJIFILM Wako Chemicals Europe). Ketone bodies were quantified with the Ketone Body Assay Kit (MAK134, Sigma Aldrich). TAG concentration was determined using the Triglyceride Assay Kit (ab65336, Abcam) and plasma glycerol level was measured using the Glycerol Assay Kit (MAK117, Sigma Aldrich). All assays were performed according to the manufacturers’ instructions.

### Cell culture and differentiation

Primary human skeletal muscle cells were isolated from vastus lateralis muscle biopsies of healthy individuals (aged 20–65 years), with protocols approved by the ethics committee in Stockholm. Myoblasts were cultured in growth medium and used between passages 6 and 9. Differentiation to myotubes was induced using specialised medium containing DMEM, Medium 199 and various supplements as described [25]. Mouse C2C12 and rat L6 myocytes were grown as described previously [25]. Differentiation was monitored microscopically, and mycoplasma contamination was routinely checked by PCR. For all cells experiments, I-BOP was diluted in DMSO.

### Glucose uptake and glycogen synthesis in cultured myotubes

Myotubes were incubated with agonists in serum-free, low-glucose DMEM (5.5 mmol/l glucose) for 4 h, as described previously [25]. Glucose uptake was measured by adding 2-deoxy-d-[1,2-^3^H]glucose (37 MBq/ml) and 10 µmol/l unlabelled 2-deoxy-d-glucose for 15 min. Cell lysate was analysed using a liquid scintillation counter and normalised to protein content. For antagonist experiments, myotubes were pre-incubated with seratrodast (10 µmol/l) or DMSO for 30 min before adding I-BOP (100 nmol/l) for 2 h prior to the uptake assay. To measure glycogen synthesis, myotubes were stimulated with insulin (100 nmol/l) for 30 min. Thereafter, d-[U-^14^C]glucose was added for the final 90 min for measurement of glucose incorporation to glycogen. Glycogen was precipitated by addition of 99% ethanol and incubated overnight at −20°C. Glycogen pellets were collected by centrifugation for 15 min at 10, 000 *g*, washed once with 70% ethanol, and resuspended in 0.3 ml distilled water. Thereafter, [^14^C]glycogen was counted in a liquid scintillation counter and normalised to protein content.

### Glucose and palmitate oxidation in cultured myotubes

Metabolic assays in myotubes were performed as described previously [25]. To measure glucose oxidation, myotubes were incubated with d-[U-^14^C]glucose (74 MBq/ml) for 4 h at 37°C, with or without FCCP (2 µmol/l). Thereafter, [^14^C]CO_2_ was captured in NaOH and quantified by liquid scintillation counting. To measure fatty acid oxidation, myotubes were incubated with [9,10-^3^H]palmitate (0.078 µmol/l) for 4 h, with or without I-BOP (50 µmol/l). Oxidation products were isolated using charcoal buffer, measured by liquid scintillation counting and normalised to protein content.

### Glucose uptake, glucose oxidation and glycogen synthesis in isolated muscle

EDL and soleus muscles isolated from mice were incubated ex vivo with I-BOP, Cicaprost, or Prostaglandin E2 for up to 3 hours. Glucose transport was measured using radiolabelled 2-deoxy-d-glucose. Glucose oxidation and glycogen synthesis were measured using radiolabelled D-[^14^C(U)]-glucose. Details of the experimental methods are available in ESM Methods.

### Protein extraction and immunoblot analysis

Cells were homogenised in ice-cold buffer (10% glycerol, 5 mmol/l sodium pyrophosphate, 137 mmol/l NaCl, 2.7 mmol/l KCl, 1 mmol/l MgCl_2_, 20 mmol/l Tris, pH 7.8, 1% Triton X-100, 10 mmol/l sodium fluoride, 1 mmol/l EDTA, 0.2 mmol/l phenylmethylsulfonyl fluoride, 1 g/ml aprotinin, 1 g/ml leupeptin, 0.5 mmol/l sodium vanadate, 1 mmol/l benzamidine, 1 mol/l microcystin). Protein concentration was determined using the bicinchoninic acid (BCA) assay. Samples were prepared in Laemmli buffer and separated by SDS-PAGE on Criterion XT Bis-Tris Gels, then transferred to PVDF membranes. Membranes were blocked in 5% non-fat milk, then incubated overnight with primary antibodies. Equal loading of protein was verified by Ponceau staining. Primary antibodies were as follows: phospho-protein kinase A (PKA) substrates (1:1000, Cell Signaling 9624), phospho-filamin A (Ser2152, 1:1000, Cell Signaling 4761), phospho-cofilin (Ser3, 1:1000, Cell Signaling 3311), phospho-Ezrin (Thr567)/Radixin (Thr564)/Moesin (Thr558) (1:1000, Cell Signaling 3141), phospho-Akt (Ser473, 1:1000, Cell Signaling 4060), phospho-AS160 (T642, 1:1000, Cell Signaling 8881), phospho-AMPK alpha (Thr172, 1:1000; Cell Signaling 2531) and phospho-acetyl-CoA carboxylase (ACC, Ser79, 1:1000, Cell Signaling 3661). After secondary antibody (Goat Anti-Rabbit IgG (H + L)-HRP Conjugate, BioRad, 1706515) incubation, proteins were visualised using enhanced chemiluminescence and quantified by densitometry.

### Gene expression analysis

RNA was extracted from cells using the EZNA Total RNA Kit and quantified by spectrophotometry. cDNA was synthesised from 0.1–1 µg RNA using random hexamers and the High-Capacity cDNA Reverse Transcription Kit. Quantitative real-time PCR (qPCR) was performed on a StepOnePlus system using a custom qPCR array with primers for 380 G-protein-coupled receptors and four housekeeping genes. Relative gene expression was calculated using the ${2}^{{-\Delta \Delta \mathrm{C}}_{\mathrm{t}}}$ method using the geometric mean of all genes in the array as the reference. Microarray analysis was conducted on Affymetrix HTA 2.0 arrays at the Bioinformatics and Expression Analysis (BEA) Core Facility at Karolinska Institutet. Data were normalised using robust multi-array (RMA) averaging and annotated with ENSEMBL gene symbols. Differentially expressed genes were identified using limma package’s empirical Bayes statistics. Gene set enrichment analysis (GSEA) was performed using clusterProfiler [26–28]. Protein–protein interaction networks were constructed using the STRING database (https://string-db.org/, accession 2023-05-16). To investigate upstream transcriptional regulators, enrichment analyses were performed using EnrichR (https://maayanlab.cloud/Enrichr/, accession 2024-12-07) against the ENCODE and ChEA consensus transcription factors [29] using significant genes (FDR <0.001) as input.

### Statistics

Statistical analyses were performed using R 4.3.0 (www.r-project.org). Data normality was verified using the Shapiro–Wilk test. When data were normally distributed, a *t* test or ANOVA with Tukey’s multiple comparison was used. For data not normally distributed, Wilcoxon signed-rank or Kruskal–Wallis test was used. Sample size and statistical tests are described in figure captions and statistical results for oxylipins are presented in the ESM dataset. Statistical significance was defined as *p*<0.05. In all box and whisker plots, boxes represent the 25th–75th percentiles with the median indicated by a horizontal line; whiskers extend to the most extreme values within 1.5× IQR; points beyond the whiskers represent outliers.

## Results

### Exercise alters oxylipin production

**Fig. 1:**
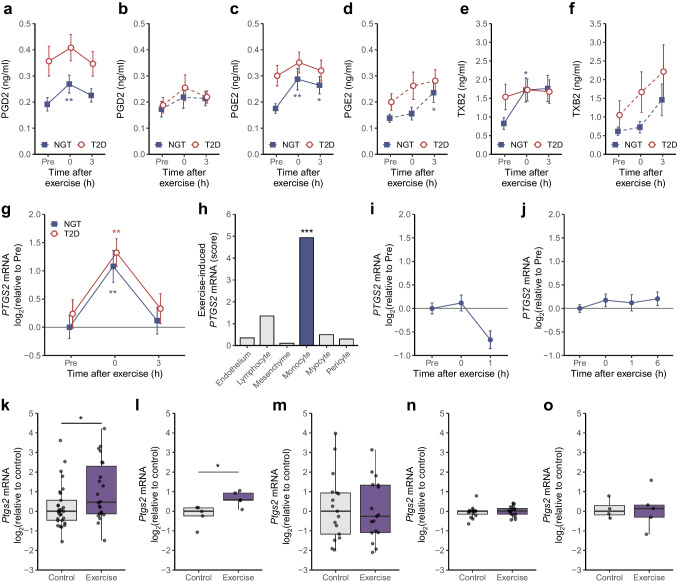
Exercise acutely increases the production of arachidonic acid metabolites. (**a**–**f**) Human blood samples were collected before (Pre), immediately after exercise (0 h) and at 3 h into the recovery period. Oxylipins were measured by MS. Plasma levels of PGD2 (**a**, **b**), PGE2 (**c**, **d**) and TXB2 (**e**, **f**) in men ([**a**, **c**, **e]**; normal glucose tolerance *n*=17, type 2 diabetes *n*=18) and women ([**b**, **d**, **f]**; normal glucose tolerance *n*=11, type 2 diabetes *n*=14). Data are mean ± SE. *p<0.05, **p<0.01 vs Pre (multi-level linear model with Benjamini–Hochberg FDR correction). Analysis with linear modelling (exercise × sex × type 2 diabetes) revealed no statistically significant interactions. (**g**) *PTGS2* mRNA in skeletal muscle tissue before and after exercise in men with type 2 diabetes or normal glucose tolerance (GSE202295). Data are mean ± SE (NGT *n*=17, type 2 diabetes *n*=20). **p<0.01 vs pre-exercise (analysed with linear modelling [exercise × sex × type 2 diabetes]). (**h**) *PTGS2* mRNA induced in skeletal muscle 3 h after a single exercise bout, analysed by single-cell RNA-seq ([15]; GSE214544). Data are presented as the score; FDR is provided in the original publication [15], ***FDR <0.001 vs pre-exercise. (**i**, **j**) *PTGS2* expression before and after exercise in human whole blood (**i**) and PBMCs (**j**) from publicly available datasets. Data are normalised to pre-exercise *PTGS2* levels and shown as mean ± SE. Statistical comparisons at each time point vs pre-exercise were conducted using the Kruskal–Wallis test followed by Tukey’s HSD post hoc analysis. (**k**–**o**) *Ptgs2* mRNA expression in mouse skeletal muscle (**k**), heart (**l**), inguinal white adipose tissue (**m**), liver (**n**) and brown adipose tissue (**o**) in control and exercised animals from publicly available datasets. Tissues were collected immediately at the end of the exercise bout. Data are shown as box and whisker plots with individual data points. *p<0.05 (ANOVA adjusted for GEO dataset as a co-variate). NGT, normal glucose tolerance; Pre, pre-exercise; T2D, type 2 diabetes

Using metabolomics targeted to measure oxylipins (ESM Methods), we investigated the impact of acute exercise on production of eicosanoids (arachidonic acid derivatives), as well as the effect of biological sex or type 2 diabetes. Among the 18 analysed arachidonic metabolites, 15 were modulated by exercise, including four eicosanoids primarily synthesised by cyclooxygenase (COX) enzymes: prostaglandin D_2_ (PGD2), prostaglandin E_2_ (PGE2), 12-hydroxyheptadecatrienoic acid (12-HHTrE) and thromboxane B_2_ (TXB2) (ESM dataset). Exercise altered circulating prostanoid concentrations over time. Plasma levels of PGD2, PGE2 and TXB2 increased immediately post exercise (Fig. 1a–f). No significant differences in prostanoid levels were found between sexes or due to type 2 diabetes status (Fig. 1a–f). Women appeared to exhibit a delayed and gradual increase in PGE2 and TXB2 levels compared with men but these differences did not reach statistical significance. Collectively, these findings demonstrate that exercise elevates circulating prostanoid levels independently of sex and type 2 diabetes status.

To elucidate the molecular mechanisms underlying these observations, we examined COX enzyme expression. mRNA levels of *PTGS2* (encoding COX-2) were elevated immediately post exercise in skeletal muscle, exhibiting marked increases compared with both the baseline and 3 h recovery period (Fig. 1g). Single-cell analysis of post-exercise skeletal muscle populations [15] revealed that *PTGS2* mRNA upregulation was specifically localised to resident monocyte/macrophage populations within the muscular microenvironment (Fig. 1h).

In contrast to the skeletal muscle-specific response, *PTGS2* mRNA expression remained unchanged in whole blood (Fig. 1i) and PBMCs (Fig. 1j) following acute exercise. Complementary findings in male mice demonstrated *Ptgs2* mRNA upregulation in skeletal and cardiac muscle immediately post exercise (Fig. 1k, l), with no changes observed in liver or in white or brown adipose tissue (Fig. 1m–o). TXB2 levels were not altered in plasma from male mice following an acute bout of exercise (ESM Fig. 1). Collectively, these findings indicate that resident monocytes/macrophages within human skeletal muscle respond to exercise by initiating prostanoid production. This localised cellular response may mediate specific muscular effects with potential systemic implications for metabolic and inflammatory processes.

### The thromboxane receptor agonist I-BOP differentially affects glucose metabolism in myocytes and skeletal muscle

**Fig. 2:**
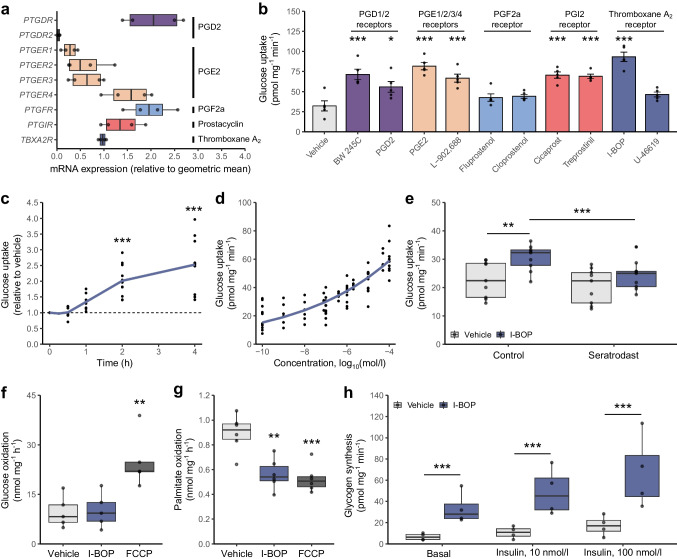
Prostanoid agonists alter skeletal muscle metabolism. (**a**) mRNA expression of prostanoid receptors in primary human skeletal myotubes was measured using a custom RT-qPCR array. Results are expressed relative to the geometric mean of all genes in the array and shown as box and whisker plots, *n*=4. (**b**) Human primary myotubes were stimulated with 50 µmol/l prostanoid receptor agonists for 4 h and glucose uptake was measured with radiolabelled 2-deoxyglucose. Results are shown as box and whisker plots (*n*=5). *p<0.05, ***p<0.001 vs vehicle (Kruskal–Wallis with Tukey’s HSD test). (**c**) Human primary myotubes were stimulated with 50 µmol/l I-BOP for up to 4 h. Glucose uptake was measured with radiolabelled 2-deoxyglucose. Results are normalised to vehicle controls and are shown as mean and individual data points. ***p<0.001 vs vehicle (one-way ANOVA with pairwise comparisons using Tukey’s HSD test). (**d**) Human primary myotubes were stimulated with increasing concentrations of I-BOP for 4 h. Glucose uptake was measured with radiolabelled 2-deoxyglucose. Results are shown as mean and individual data points (*n*=9). (**e**) Human primary myotubes were stimulated with 100 nmol/l I-BOP for 4 h in the presence of 10 µmol/l thromboxane receptor antagonist seratrodast. Results are shown as box and whisker plots (*n*=8). **p<0.01, ***p<0.001 (two-way ANOVA [I-BOP, seratrodast] with Tukey’s post-test analysis). (**f**) Human primary myotubes were stimulated with 50 µmol/l I-BOP for 4 h. Glucose oxidation was measured by the oxidation of [^14^C]glucose into CO_2_. FCCP was used as a positive control. Results are shown as box and whisker plots (*n*=5). **p<0.01 vs vehicle (Kruskal–Wallis with Tukey’s HSD test). (**g**) Human primary myotubes were incubated with 50 µmol/l I-BOP for 4 h. Palmitate oxidation was measured by the oxidation of [^3^H]palmitate into H_2_O. FCCP was used as a positive control. Results are shown as box and whisker plots (*n*=6). **p<0.01, ***p<0.001 vs vehicle (Kruskal–Wallis with Tukey’s HSD test). (**h**) Human primary myotubes were incubated with 50 µmol/l I-BOP for 4 h, followed by stimulation with 100 nmol/l insulin for 30 min. Glycogen synthesis was measured by the incorporation of [^14^C]glucose into glycogen as described in Methods. Results are shown as box and whisker plots (*n*=4). ***p<0.001 (two-way ANOVA [I-BOP, insulin] with Tukey’s HSD test). PGF2α, prostaglandin F_2α_, PGI2, prostaglandin I_2_

Human primary myotubes express detectable mRNA levels of prostanoid receptors (Fig. 2a), suggesting a potential responsiveness to prostanoid signalling. Agonists of thromboxane receptors, including PGD2, prostaglandin I_2_ and PGE2, increased glucose uptake (Fig. 2b). Conversely, activation of the prostaglandin F_2α_ receptor did not affect glucose uptake (Fig. 2b). In rat L6 and mouse C2C12 myotubes, of the prostanoid receptor agonists used, only the thromboxane receptor agonist I-BOP increased glucose uptake (ESM Fig. 2a, b). As I-BOP was the only agonist that consistently stimulated glucose uptake across all cell models examined (C2C12, L6 and human primary myotubes), this compound was selected for in vitro and in vivo mechanistic analyses.

I-BOP increased glucose uptake in primary human myotubes, with effects observed as early as 1 h after incubation and effects becoming more pronounced at 4 h (Fig. 2c). This response exhibited a dose-dependent relationship (Fig. 2d) up to a maximum of 2.5-fold. Pre-treatment with the thromboxane receptor antagonist seratrodast attenuated I-BOP-induced glucose uptake (Fig. 2e), confirming the specificity of thromboxane receptor activation in modulating skeletal muscle glucose metabolism. While I-BOP was without effect on glucose oxidation (Fig. 2f), it decreased fatty acid oxidation (Fig. 2g) and enhanced glycogen synthesis by 430% (Fig. 2h). There was no interaction between I-BOP and insulin with regard to glycogen synthesis, suggesting that I-BOP increases glucose metabolism through insulin-independent signalling pathways.

### Thromboxane receptor activation activates cytoskeletal remodelling

**Fig. 3:**
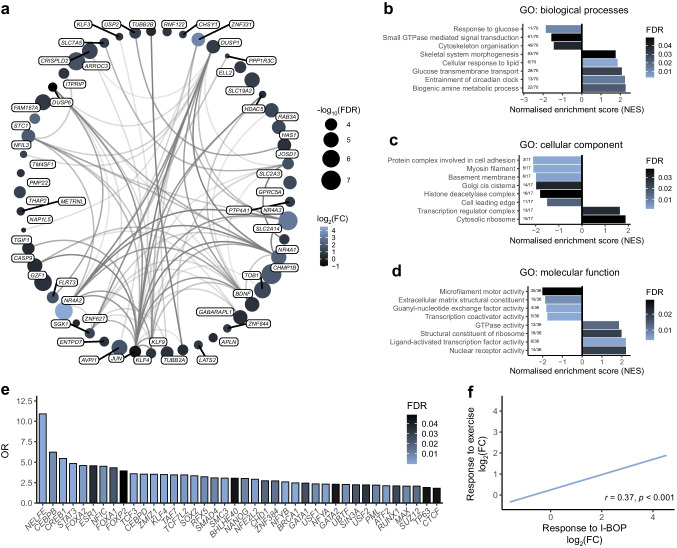
Stimulation of the thromboxane receptor activates transcriptional and cytoskeletal remodelling pathways. RNA extracted from primary human skeletal muscle cells exposed to I-BOP (50 µmol/l, 2 h) was analysed using the human HTA 2.0 microarray. (**a**) Known interactions between significant genes (FDR <0.001) were plotted using StringDB. (**b**–**d**) GSEA was performed on genes ranked on log_2_(fold change) using the Gene Ontology database. Representative pathways and FDRs are shown. (**e**) Transcription factor enrichment using EnrichR. (**f**) Comparison of the transcriptomic response to I-BOP with the transcriptomic response to acute aerobic exercise from the MetaMEx database. Spearman correlation. FC, fold change; GO, Gene Ontology

Transcriptomic analysis of human skeletal muscle cells following thromboxane receptor activation revealed that I-BOP predominately regulated genes associated with the NR4A family of transcription co-activators and the transcription factor JUN (Fig. 3a). GSEA using the Gene Ontology database suggested activation of metabolic regulation, transcriptional modulation and cytoskeleton remodelling (Fig. 3b). Cellular component analysis demonstrated that the transcriptomic response to I-BOP included modulation of structural and functional elements, particularly ribosomes and cytoskeleton (Fig. 3c). Additionally, genes associated with ‘molecular functions’ activated by I-BOP encompassed GTPase activity, microfilament motor activity and transcriptional regulation (Fig. 3d). Transcription factor motif enrichment analysis of the top 100 I-BOP-responsive genes, ranked by FDR, highlighted the involvement of G protein-coupled receptor (GPCR)-responsive factors, notably *CREB1* and *STAT3*, alongside several non-canonical transcription factors (Fig. 3e). These findings suggest that I-BOP concurrently activates five primary molecular signalling pathways: cAMP–PKA, JAK–STAT3, MAPK, PI3K and Rho-GTPases.

The transcriptomic response induced by I-BOP activated several exercise-responsive genes, including *NR4A3*, *IL6*, *JUNB* and *KLF4*. Using the MetaMEx database [30], we compared the gene expression changes with the transcriptomic response of human skeletal muscle to exercise and inactivity. Notably, genes responsive to thromboxane receptor activation (FDR <0.05) showed a strong correlation (*r*=0.37, *p*<0.001) with the transcriptional response to acute exercise (Fig. 3f).

**Fig. 4:**
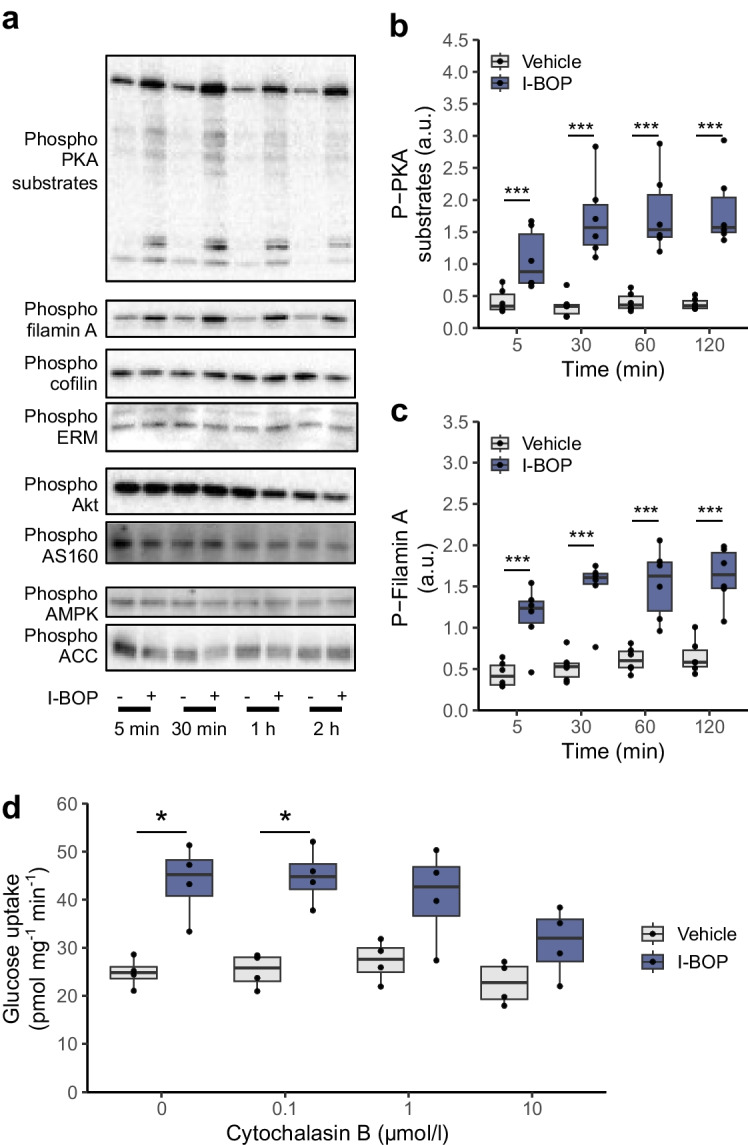
Thromboxane receptor promotes actin remodelling. Human primary myotubes were treated with 50 µmol/l I-BOP for up to 4 h. (**a**) Representative blots of phosphorylated PKA substrates, filamin A, cofilin, ezrin/radixin/moesin proteins, Akt, AS160, AMPK and ACC. (**b**, **c**) Quantification of the phosphorylation of PKA substrates and filamin A. Results are shown as box and whisker plots (*n*=6). ***p<0.001 (two-way ANOVA [I-BOP, time] with Tukey’s post hoc comparison). (**d**) Glucose uptake in L6 myotubes in response to 50 µmol/l I-BOP in the presence of increasing concentrations of cytochalasin B (0–10 µmol/l) for 4 h. Results are shown as box and whisker plots (*n*=4) *p<0.05 (two-way ANOVA [I-BOP, cytochalasin B] with Tukey’s post hoc comparison). a.u., arbitrary densitometry units

Biochemical validation confirmed the transcriptional pathway enrichment. Stimulation of human primary muscle cells with I-BOP activated PKA, evidenced by the phosphorylation of multiple PKA substrates (Fig. 4a, b). In line with the non-additive effect of I-BOP with insulin on glucose incorporation into glycogen (Fig. 2h) and the absence of an increase in substrate oxidation (Fig. 2f, g), I-BOP had no effect on phosphorylation of protein kinases involved in the Akt–AS160 or AMPK–ACC pathways (Fig. 4a). Actin remodelling was further substantiated by the phosphorylation of filamin A (Fig. 4a, c) but not cofilin or ezrin/radixin/moesin (ERM) proteins (Fig. 4a). Blockage of cytoskeletal trafficking with cytochalasin B blocked I-BOP-induced glucose uptake in a dose-dependent manner (ANOVA *p*=0.052, Fig. 4d), reinforcing a possible role for cytoskeletal remodelling in the metabolic response to thromboxane receptor stimulation.

### Thromboxane receptor agonist I-BOP modulates whole-body glucose metabolism in male mice

**Fig. 5:**
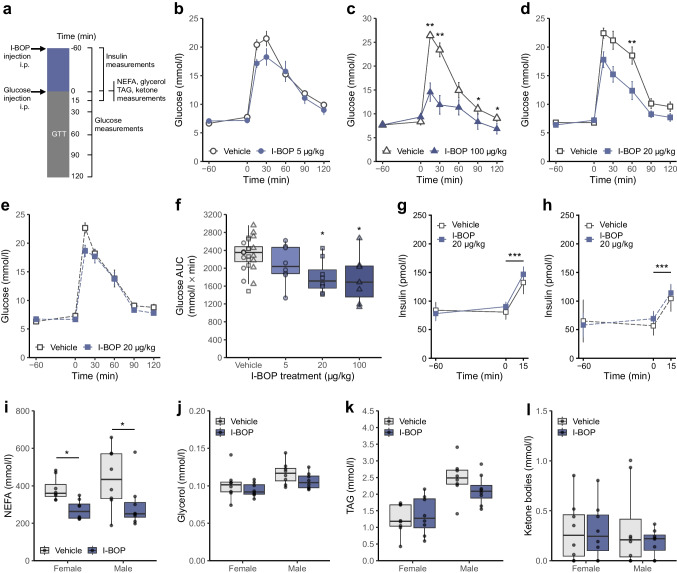
Thromboxane stimulation in vivo improves glucose tolerance. (**a**) Schematic representation of the glucose tolerance protocol with timing of measurements. (**b**) GTT in male mice after i.p. injection of 5 µg/kg I-BOP. Results are shown as mean ± SEM (*n*=8). Analysed by two-way ANOVA (I-BOP, glucose) with Tukey’s post hoc comparison. (**c**) GTT in male mice after i.p. injection of 100 µg/kg I-BOP. Results are shown as mean ± SEM (vehicle *n*=8, I-BOP *n*=7). *p<0.05, **p<0.01 vs vehicle (two-way ANOVA [I-BOP, glucose] with Tukey’s post hoc comparison). (**d**, **e**) GTT in male (**d**) and female (**e**) mice after i.p. injection of 20 µg/kg I-BOP. Results are shown as mean ± SEM (*n*=8). **p<0.01 vs vehicle (three-way ANOVA [I-BOP, glucose, sex] with Tukey’s post hoc comparison). (**f**) Glucose concentration AUC during GTTs in male mice at 5, 20 and 100 µg/kg I-BOP (presented in **b**, **c** and **d**, respectively). Results are shown as box and whisker plots (*n*=7 or 8). *p<0.05 for I-BOP vs corresponding vehicle group within each cohort of animals (individual *t* tests). (**g**, **h**) Insulin levels in male (**g**) and female (**h**) mice during the GTT. Results are shown as mean ± SEM (*n*=8). ***p<0.001 (two-way ANOVA [I-BOP, glucose] with Tukey’s post hoc comparison). (**i**–**l**) NEFA (**i**), glycerol (**j**), TAG (**k**) and ketone levels (**l**) 1 h after i.p. injection of 20 µg/kg I-BOP. Results are shown as box and whisker plots (*n*=8). *p<0.05 vs vehicle (two-way ANOVA [I-BOP, sex] with Tukey’s post hoc comparison)

Considering that there are potential parallels between acute exercise and thromboxane receptor activation, including enhanced glucose uptake in myotubes and exercise-like transcriptomic profiles, we investigated the systemic metabolic effects of a thromboxane agonist on glucose tolerance in mice (Fig. 5a). Initial experiments with a low dose of I-BOP (5 µg/kg) demonstrated modest, non-significant improvements in glucose control observable 15 min post glucose injection in male mice (Fig. 5b). A higher dose (100 µg/kg) enhanced glucose tolerance (Fig. 5c); however, this dose was accompanied by mild adverse effects, including diarrhoea and reduced ambulatory activity. We identified an optimal dose of 20 µg/kg, which was well tolerated and produced a marked improvement in glucose tolerance in male mice (Fig. 5d). Interestingly, this dose elicited comparatively attenuated effects in female mice (Fig. 5e). The glucose tolerance response exhibited a clear dose-dependent relationship (Fig. 5f), while circulating insulin levels were not affected in either male (Fig. 5g) or female (Fig. 5h) mice. Mice exposed to I-BOP exhibited reduced circulating NEFA levels (Fig. 5i), without changes in glycerol, TAG or ketone bodies (Fig. 5j–l).

Ex vivo experiments in isolated skeletal muscle (ESM Methods) yielded results that differed from our in vitro and in vivo findings. I-BOP did not increase glucose uptake in extensor digitorum longus (EDL) or soleus muscle (ESM Fig. 3a–d), although it enhanced glucose oxidation in EDL and reduced glycogen synthesis in both muscles (ESM Fig. 3e–h). Other receptor agonists had no metabolic effects ex vivo, and glucose uptake remained unchanged after in vivo I-BOP injection when muscles were subsequently incubated ex vivo (ESM Fig. 3i, j). The results obtained from our ex vivo model contrast with our in vitro findings of increased glucose uptake in cultured skeletal muscle cells (Fig. 2) and in vivo findings of improved glucose tolerance (Fig. 5) following I-BOP exposure. This discrepancy may be attributable to elevated stress and inflammation responses in skeletal muscle maintained ex vivo (ESM Fig. 4), which could interfere with thromboxane signalling. Therefore, we focused on characterising thromboxane-induced metabolic effects in vivo.

**Fig. 6:**
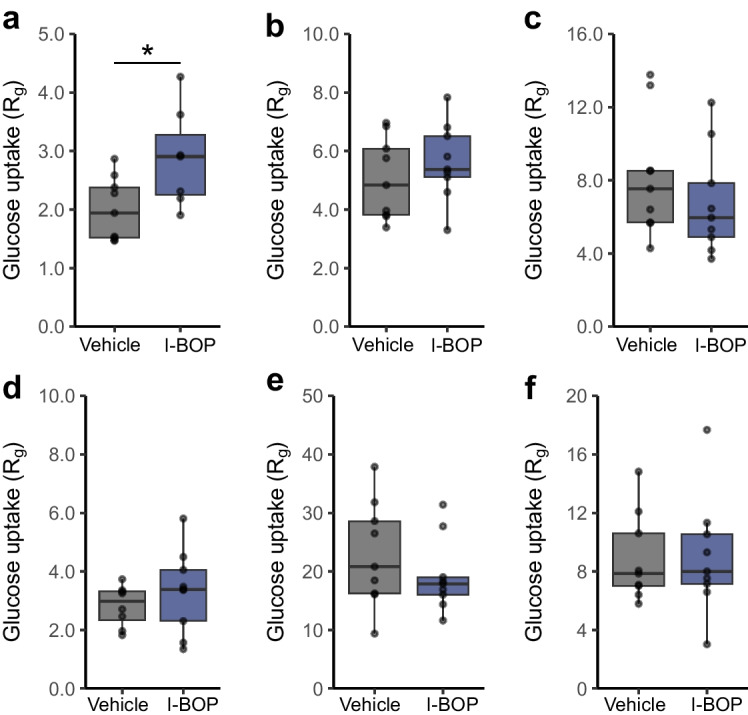
Thromboxane stimulation in vivo improves glucose uptake in skeletal muscle. Rate of glucose uptake (Rg) measured in EDL of male mice as described in Methods (**a**, vehicle *n*=9, I-BOP *n*=7), tibialis anterior (**b**, vehicle and I-BOP *n*=9) and soleus (**c**, vehicle and I-BOP *n*=9) muscles, liver (**d**, vehicle *n*=8, I-BOP *n*=9), brown adipose tissue (**e**, vehicle and I-BOP *n*=9) and epididymal adipose tissue (**f**, vehicle and I-BOP *n*=9). Results are shown as box and whisker plots. *p<0.05 vs vehicle (individual *t* tests)

In vivo glucose uptake was assessed using an i.v. bolus of 2-[^14^C]deoxyglucose co-administered with glucose and I-BOP (20 µg/kg) to assess glucose uptake (ESM Fig. 5). Tissue-specific analysis revealed that I-BOP increased glucose uptake in EDL muscle by 43% (Fig. 6a), with a comparable, although non-significant, increase observed in the tibialis anterior (Fig. 6b). No alterations in glucose uptake were detected in the soleus, liver, brown adipose tissue or white adipose tissue (Fig. 6c–f).

**Fig. 7:**
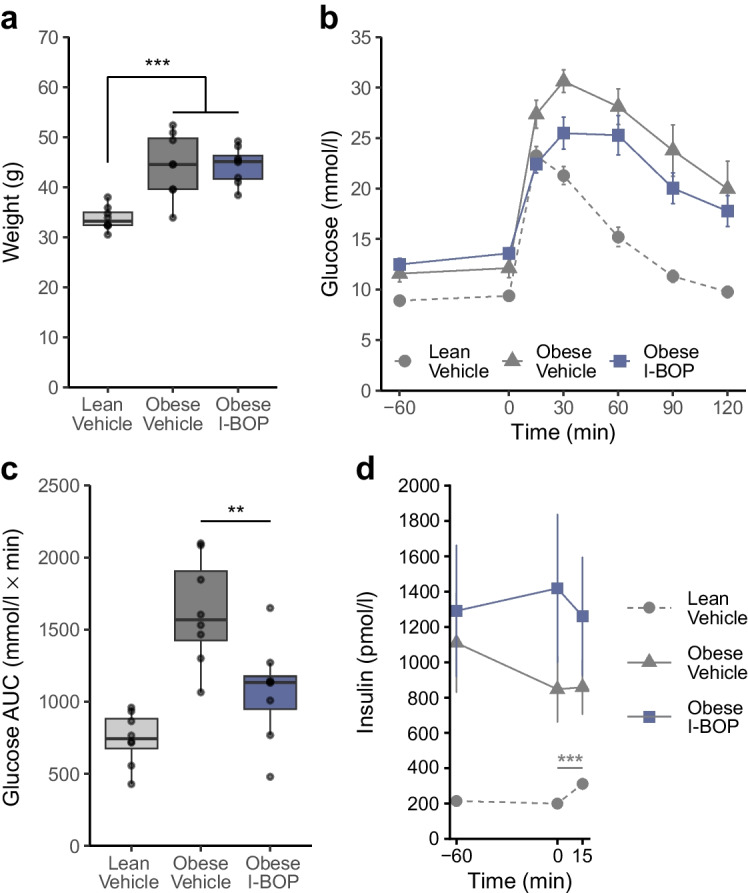
Thromboxane stimulation in vivo improves glucose tolerance in obese mice. (**a**) Body weight of male mice after 12 weeks of exposure to a control diet, or to a high-fat diet to induce obesity and insulin resistance. Results are shown as box and whisker plots (*n*=9 BAT, eWAT, soleus and TA; *n*=9 EDL vehicle; *n*=7 EDL I-BOP; *n*=8 liver vehicle; *n*=9 liver I-BOP). ***p<0.001 vs lean vehicle (individual *t* test). (**b**) GTTs in male mice exposed to a high-fat diet and treated acutely with 20 µg/kg I-BOP by i.p. injection. Results are shown as mean ± SEM, *n*=8 (analysed by two-way ANOVA [I-BOP, glucose]). (**c**) Glucose concentration AUC during the GTT. Results are shown as box and whisker plots (*n*=8). **p<0.01 for I-BOP vs vehicle in high-fat-diet-fed mice (individual *t* test). (**d**) Insulin levels in male mice during the GTT. Results are shown as mean ± SEM (*n*=8). ***p<0.001 for I-BOP vs vehicle in high-fat-diet-fed mice (two-way ANOVA [I-BOP, glucose] with Tukey’s post hoc comparison)

To further explore the therapeutic potential of thromboxane stimulation, we examined the effect of I-BOP on glucose metabolism in a model of diet-induced metabolic dysfunction. In male mice subjected to a high-fat diet to induce obesity (Fig. 7a), I-BOP administration improved glucose tolerance (Fig. 7b, c) without affecting insulin levels (Fig. 7d).

## Discussion

Exercise-induced inflammation is a transient physiological response that affects skeletal muscle adaptation and metabolic regulation. Exercise increases COX abundance, activity [31] and prostanoid production [18] in healthy human skeletal muscle. Our data corroborate those findings, with induction of COX-2 in skeletal muscle and prostanoid production immediately following acute exercise in individuals with normal glucose tolerance, and we found a similar response in individuals with type 2 diabetes. Notably, we found *PTGS2* specifically localised to monocyte/macrophage populations within human skeletal muscle, indicating that these immune cells are the primary source of exercise-induced prostanoid production. Since COX-2 was not induced in circulating cells in response to exercise, plasma TXB2 levels likely reflect skeletal muscle-generated thromboxane A_2_. This localised inflammation, with thromboxane accumulation in the extracellular environment, may mediate acute exercise effects through immune-skeletal muscle cell crosstalk.

Distinct patterns of thromboxane levels emerged when comparing individuals with and without type 2 diabetes and comparing sexes. Women with type 2 diabetes exhibited sustained elevated thromboxane levels post-exercise, while men demonstrated consistently elevated baseline levels that were unaffected by exercise. These findings are consistent with prior reports of increased thromboxane biosynthesis in type 2 diabetes [32] and elevated circulating PGE2 levels compared with healthy, normal weight, or obese individuals [33]. The persistently high PGE2 levels in type 2 diabetes may reflect the chronic low-grade inflammation characteristic of metabolic diseases, and correspond with impaired inflammatory responses in skeletal muscle [6]. Compared with the transient increases in PGE2 and PGD2, plasma TXB2 levels remained elevated for up to 3 h after exercise. This pattern aligns with previous observations of plasma prostanoid elevation in young healthy men following resistance exercise, an effect that was entirely suppressed by ibuprofen [16]. Thromboxane appears to be produced more consistently than other prostaglandins, returning to baseline 24 h post-exercise, suggesting a role in both immediate and delayed skeletal muscle remodelling [16, 18].

Transcriptomic analysis in skeletal muscle cells exposed to a thromboxane receptor agonist revealed the activation of genes associated with myocyte structural constituents, oxidative stress response and metabolic pathways, suggesting a multifaceted role for thromboxane in exercise-induced remodelling processes. At the molecular level, cytoskeletal remodelling plays a critical role in regulating glucose uptake by facilitating GLUT translocation [34]. Our transcriptomic and signalling analyses revealed that thromboxane receptor stimulation leads to PKA activation and filamin A phosphorylation. This mechanism parallels PKA activation downstream of β_2_-adrenergic receptors, known to induce actin remodelling and promote skeletal muscle glucose uptake [35]. Although the thromboxane receptor canonically signals through Gq/11 and G12/13 proteins, these signal transducers can stimulate adenylate cyclase independently, leading to increased intracellular cAMP and subsequent PKA activation [36]. Moreover, exercise induces filamin A phosphorylation [37] and dynamic actin rearrangement [38]. Thus, PKA activation and actin remodelling could therefore contribute to the enhanced GLUT4 translocation and glucose uptake.

Our findings add a new dimension to the understanding of thromboxane biology in skeletal muscle. Previous studies have primarily focused on thromboxane signalling in vascular and inflammatory contexts, where thromboxane receptor activation is typically associated with vasoconstriction, platelet activation and proinflammatory responses [12, 32]. In contrast, very little is known about its metabolic actions. Here, we show that the thromboxane receptor agonist I-BOP robustly stimulated glucose uptake in rat, mouse and human myotubes in vitro, indicating a conserved mechanism across species. In vivo, I-BOP enhanced glucose uptake in oxidative skeletal muscles in mice, without affecting hepatic or adipose metabolic responses. Importantly, the ability of I-BOP to enhance glucose metabolism even in a model of insulin resistance suggests that thromboxane signalling may engage alternative pathways that remain functional when classical insulin signalling is impaired, a phenomenon observed for instance in response to acute exercise [39]. Together, these findings suggest that locally targeting the thromboxane receptor in skeletal muscle can promote glucose uptake through mechanisms that bypass pathways commonly impaired in metabolic disease.

Our study indirectly suggests that inhibiting COX-2 may negatively impact the metabolic response to exercise by reducing thromboxane levels. Emerging evidence suggests complex interactions between non-steroidal anti-inflammatory drugs (NSAIDs) and metabolic processes. In individuals with type 2 diabetes, NSAID use has been associated with improved glucose tolerance [40] and a causal link between ibuprofen and hypoglycaemia has been established [41]. However, the effects of COX-2 inhibition on skeletal muscle remodelling remain contentious [10]. While NSAIDs may reduce exercise-induced muscle damage and alleviate soreness, they may also impair satellite cell activity, a process essential for effective muscle repair. The variability in outcomes suggests that the effects of COX-2 inhibition on skeletal muscle remodelling may be influenced by multiple factors, including exercise type, drug administration timing and individual physiological differences.

### Limitations of the study

Differential responses across prostanoid agonists highlight the complexity of metabolic signalling pathways, necessitating further investigation into receptor-specific mechanisms. Sex-dependent differences in thromboxane signalling represent both a limitation and a scientific opportunity. The lack of improvement in glucose tolerance in female mice suggests that hormonal and immune modulators profoundly influence prostanoid-mediated regulation of metabolism. These findings expose a critical gap in understanding sex-specific metabolic responses, historically underexplored in exercise research. The concentration-dependent side effects of thromboxane receptor agonists emphasise the challenges of systemic intervention, highlighting the need for targeted therapeutic approaches that minimise systemic complications.

### Conclusion

Our study reveals a novel molecular pathway linking exercise-induced prostanoid production to metabolic adaptation. By elucidating the role of thromboxane in glucose uptake and glycogen accumulation, we challenge existing paradigms of the canonical role of thromboxane and propose new therapeutic strategies for metabolic disorders. Future research should focus on developing targeted prostanoid-based approaches that optimise exercise’s metabolic benefits while minimising potential side effects.

## Supplementary Information

Below is the link to the electronic supplementary material.

ESM1 (PDF 552 KB)

ESM2 (XLSX 220 KB)

## Data Availability

Data supporting the findings shown in Fig. 1 are available within the paper and its ESM. Other data are available from the corresponding author on reasonable request.

## Abbreviations

ACC: Acetyl-CoA carboxylase
COX: Cyclooxygenase
EDL: Extensor digitorum longus
FDR: False discovery rate
GEO: Gene Expression Omnibus
GSEA: Gene set enrichment analysis
NSAID: Non-steroidal anti-inflammatory drug
PBMC: Peripheral blood mononuclear cell
PGD2: Prostaglandin D_2_
PGE2: Prostaglandin E_2_
PKA: Protein kinase A
TAG: Triacylglycerol
TXB2: Thromboxane B_2_
