# Supplementary material for: Thromboxane signalling links immune activation to enhanced glucose uptake in skeletal muscle
Source: Diabetologia. 2026 Feb 20;69(6):1643–58. doi: 10.1007/s00125-026-06684-8 (PMC13109283; doi:10.1007/s00125-026-06684-8)
Supplement: Supplementary file 1 — ESM1 (PDF 552 KB) [file 125_2026_6684_MOESM1_ESM.pdf]

Methods

Acute exercise in male and female mice. Five-month-old male and female C57BL/6J mice (n = 6 per sex) were housed under standard laboratory conditions with ad libitum access to food and water. Animals underwent three treadmill acclimation sessions over one week using a motorised treadmill set to a fixed 25° incline and operated without electrical stimulation. The acclimation protocol progressed in intensity across sessions: 0 m/min (3 min), 6 m/min (3 min), 10 m/min (2 min), and 12 m/min (1 min) in session one; 0 m/min (1 min), 10 m/min (3 min), 12 m/min (2 min), and 14 m/min (2 min) in session two; and 8 m/min (3 min), 10 m/min (2 min), 12 m/min (1 min), 14 m/min (2 min), 1 min rest, 16 m/min (2 min), 1 min rest, and 18 m/min (2 min) in session three. Basal blood samples were collected from the tail vein 24 hours after the final acclimation. The following day, mice performed an acute high-intensity interval training (HiiT) session on the same treadmill. To minimise delays in post-exercise sampling, only three animals were exercised in parallel. The HiiT protocol consisted of 2 min at 8 m/min, 2 min at 10 m/min, 1 min at 12 m/min, 1 min at 14 m/min, followed by 1 min of rest, then nine intervals of 3 min at 16 m/min, each separated by 1 min of passive rest. Tail blood was collected immediately after exercise using heparinised glass capillaries, held on ice for 15 minutes, and centrifuged at 1,200 × g for 10 minutes at 4 °C. Plasma was aliquoted and stored at –80 °C until analysis.

Oxylipin analysis. After thawing samples at 4°C and vortex them for 5 seconds, a volume of 200 µL of plasma was transferred to a 12 x 75 mm Pyrex glass tube. Samples were then spiked with 10 µL of an isotopically labelled mixture of oxylipin as internal standard (**ESM Table 2**). Afterwards, samples were diluted with 800 µL of an aqueous solution of 0.1 M citric acid/0.2 M Na_2HPO_4 at pH = 5.6. Next, samples were extracted using an Extrahera^{TM} Classic automated extraction system (Biotage, Uppsala, Sweden). Extraction was performed using HLB Oasis 3cc (60mg sorbent) cartridges (Waters, Milford; PN: 186001880). Cartridges were first conditioned with 2 mL of LC-MS methanol (Optima, Fisherbrand) followed by 2 mL of Mili-Q water. Afterwards, the diluted samples were loaded onto the cartridge. This was followed by a 2 mL wash step with water:methanol (9:1, v/v). Cartridges were then dried by applying a positive pressure step with N_2. Oxylipins were finally eluted from the cartridge with 2.5 mL of LC-MS methanol and collected in a Pyrex 12x75 mm tube. Then, methanol was evaporated using a Turbovap® LV automated solvent evaporation system (Biotage, Uppsala, Sweden). Then, dried extracts were reconstituted in 80 µL of MeOH/water (6:1, v/v), vortexed and filtered through a 0.1 PVDF membrane spin filter (Amicron, Merck, Billerica, MA; PN: UFC30VV00) followed by a 3.5-minute centrifugation step at 5000 *g* at 6°C. The filtered sample solution was finally transferred to an LC-MS vial equipped with a 150 µL insert for its injection.

For TXB_2 in mice, plasma samples were extracted using protein precipitation. Briefly, after thawing samples at 4°C and vortex them for 5 seconds, a volume of 25 µL of plasma was transferred to an Eppendorf tube. Samples were then spiked with 10 µL of an isotopically labelled mixture of oxylipin as internal standard (8-fold diluted from the plasma mixture). This was followed by the addition of 150 µL of methanol. Samples were then vortexed for 5 seconds, equilibrated at 4°C for 20 minutes and finally centrifuged at 12000 g for 15 minutes. A volume of 125 µL was finally transferred to an LC-MS vial equipped with a 150 µL insert for its injection.

Samples were analysed on an ACQUITY UPLC System coupled to a Xevo® TQ-S (human plasma) or Xevo® TQ-XS (Mouse plasma) triple quadrupole system, both from Waters Corporation (Milford, MA), equipped with an electrospray ion source operating in the negative ion mode as previously described. Separation was performed on an Acquity BEH C_{18}-column (2.1 mm × 100 mm, 1.7 µm, Waters, Milford, MA, USA), with mobile phase A consisting of Milli-Q water and CH_3COOH 0.1% v/v and mobile phase B consisting of ACN:IPA (9:1). The column oven and sample manager temperatures were set to 35°C and 8°C, respectively. Gradient elution performed starting from 35% B, which was linearly increased to 40% at 2.1 min, to 42% at 3.5 min, to 50% at 5.5 min, to 72.5% at 11.5 min, and to 100% at 11.7 min. The column was then washed in 100% B for 1.8 min and re-equilibrated under initial conditions for 1.4 min. The resulting separation time was 11.5 min, for a total run time of 15 min including injection and equilibration. The injection volume was set to 7.5 µL, and the flow rate was kept at 0.425 mL/min. Details of standards used for quantification, retention time and selected reaction monitoring (SRM) transition for each oxylipin are detailed in **ESM Table 2**. For mouse plasma, only TXB_2 was quantified.

Glucose uptake in isolated muscle. Mice (15-24 weeks old) were anesthetised via intraperitoneal injection of 2.5% tribromoethanol. Extensor digitorum longus (EDL) and soleus muscles were preincubated for 30 minutes or 3 hours at 30°C in oxygenated Krebs-Henseleit bicarbonate buffer (KHB) containing 5 mM glucose, 15 mM mannitol, 5 mM HEPES, and 0.1% BSA, with or without I-BOP (10-100 µM). Glucose transport was measured by incubating muscles for 20 minutes in KHB with 1 mM cold 2-deoxy-D-glucose, [1,2-3H]-2-deoxy-D-glucose

(2.5 µCi/ml), 19 mM cold mannitol and [14C] mannitol (0.7 µCi/ml). Muscles were quickly frozen in liquid nitrogen, lysed, and analysed by scintillation counting.

Glucose oxidation and glycogen synthesis in isolated muscle. Fourteen-weeks-old C57BL/6J mice were fasted for 4 hours and anesthetised. EDL and soleus muscles were incubated with I-BOP, Cicaprost, or Prostaglandin E2 for 3 hours, with media replacement at 1.5 hours. Muscles were then incubated in rubber lead sealed wells in 2 ml of KHB buffer containing D-[^{14}C(U)]-glucose, with or without insulin (120 nM). An open top container filled with 500 µl of 1:1 mixture of Protocatechuic acid and Solvable was attached to the lid of each sealed well. After 1 h incubation, muscles were taken out from vials and snap frozen in liquid nitrogen. All vials were sealed again, and 500 µl of 1% perchloric acid was injected into incubation media through lids. Liberated CO_2 was captured by Protocatechuic acid and Solvable, then quantified by liquid scintillation counted and normalised to muscle weight. Muscles were dissolved in 1 M NaOH at 70°C and the solution neutralised with 20% trichloroacetic acid. After addition of non-labelled glycogen carrier and precipitation with ethanol, supernatant was discarded and total glycogen resuspended in water and quantified by liquid scintillation counting.

Tables

	NGT		T2D		Diabetes p-value	Sex p-value	Diabetes:sex p-value
**Sex**	Female	Male	Female	Male			
**Participants**	11	17	14	18			
**Age (years)**	62.8 ± 4.8	61.6 ± 4.7	63.1 ± 4.5	59.7 ± 5.3	0.555	0.030	0.392
**BMI (kg/m^2)**	27.7 ± 2.7	26.9 ± 2.8	28.4 ± 3.4	27.4 ± 2.7	0.404	0.238	0.946
**Waist/Hip ratio**	0.86 ± 0.05	0.96 ± 0.05	0.93 ± 0.06*	1.00 ± 0.07*	<0.001	<0.001	0.326
**Body fat (%)**	38.3 ± 6.3	27.1 ± 5.3	41.3 ± 4.9	29.7 ± 4.7	0.017	<0.001	0.882
**Fasting glucose (mmol/l)**	5.5 ± 0.5	5.2 ± 0.5	7.5 ± 2.1*	7.1 ± 1.3*	<0.001	0.249	0.641
**HbA1c (mmol/mol)**	35.5 ± 3.6	35.5 ± 3.2	48.9 ± 9.9*	48.6 ± 8.8*	<0.001	0.965	0.983
**HbA1c (%)**	5.40 ± 0.33	5.40 ± 0.29	6.62 ± 0.91*	6.60 ± 0.80*	<0.001	0.965	0.983
**HOMA-IR**	2.2 ± 0.7	1.48 ± 0.89	3.4 ± 1.5	3.5 ± 2.0*	<0.001	0.060	0.102
**Triglycerides (mmol/l)**	0.82 ± 0.21	1.00 ± 0.43	1.62 ± 0.54*	1.38 ± 0.83	<0.001	0.472	0.067
**Total cholesterol (mmol/l)**	5.0 ± 0.6	5.4 ± 0.9	4.8 ± 0.6	4.3 ± 0.9*	<0.001	0.778	0.035
**HDL cholesterol (mmol/l)**	1.76 ± 0.40	1.51 ± 0.40	1.50 ± 0.34	1.21 ± 0.32	0.005	0.005	0.824
**LDL cholesterol (mmol/l)**	2.9 ± 0.6	3.4 ± 0.8	2.6 ± 0.6	2.5 ± 0.8*	<0.001	0.205	0.084

ESM Table 1. Clinical characteristics of study participants. Data are presented as mean ± standard deviation. Group comparisons were performed using a two-way ANOVA with diagnosis (NGT vs. T2D) and sex (Female vs. Male) as factors. The reported p-values indicate main effect of diabetes status, main effect of sex and interaction effect. Post hoc comparisons were conducted using Tukey's HSD test, and values marked with * indicate $p < 0.05$ when comparing NGT vs. T2D within each sex.

Compound Name	Standard	Systematic Name	Supplier	Cat. Number	Retention time (min.)	SRM transition	Internal Standard
11,12-DiHETrE	(±)11,12-DiHETrE	11,12-dihydroxy-5Z,8Z,14Z-eicosatrienoic acid	Cayman Chemical	10007266	7.6	337.3 > 166.9	d11-11,12-DiHETrE
11-HDoHE	(±)11-HDoHE	(±)11-hydroxy-4Z,7Z,9E,13Z,16Z,19Z-docosahexaenoic acid	Cayman Chemical	33450	10.0	343.3 > 121.0	d8-12-HETE
11-HEDE	(±)11-HEDE	(±)11-hydroxy-12E,14Z-eicosadienoic acid	Cayman Chemical	37500	11.1	323.2 > 199.0	d4-9-HODE
11-HEPE	(±)11-HEPE	(±)11-hydroxy-5Z,8Z,12E,14Z,17Z eicosapentaenoic acid	Cayman Chemical	32500	8.5	317.2 > 166.7	d8-12-HETE
11-HETE	(±)11-HETE	11-hydroxy-5Z,8Z,11E,14Z-eicosatetraenoic acid	Cayman Chemical	34500	9.8	319.2 > 166.8	d8-12-HETE
12(13)-EpODE	12,13-EpODE	12,13-epoxy-9Z,15Z-octadecadienoic acid	Larodan	14-1803-16a-1	9.6	293.2 > 183.0	d4-9(10)-EpOME
12,13-DiHOME (erythro)	(±)12,13-DiHOME	(±)12,13-dihydroxy-9Z-octadecenoic acid	Cayman Chemical	10009832	6.0	313.1 > 183.1	d4-12,13-DiHOME
12,13-DiHOME (threo)	(±)12,13-DiHOME	(±)12,13-dihydroxy-9Z-octadecenoic acid	Cayman Chemical	10009832	6.5	313.1 > 183.1	d4-12,13-DiHOME
12-HEPE	(±)12-HEPE	(±)12-hydroxy-5Z,8Z,10E,14Z,17Z-eicosapentaenoic acid	Cayman Chemical	32540	8.8	317.0 > 178.8	d8-12-HETE
12-HETE	(±)12-HETE	(±)12-hydroxy-5Z,8Z,10E,14Z-eicosatetraenoic acid	Cayman Chemical	34550	10.0	319.1 > 179.0	d8-12-HETE
12-HHTrE	12(S)-HHTa	12S-hydroxy-5Z,8E,10E-heptadecatrienoic acid	Cayman Chemical	34590	7.3	279.2 > 179.0	d4-13-HODE
12-KETE	12-oxo-ETE	12-oxo-5Z,8Z,10E,14Z-eicosatetraenoic acid	Cayman Chemical	34580	10.1	317.2 > 153	d6-5-KETE
13-HODE	(±)13-HODE	(±)-13-hydroxy-9Z,11E-octadecadienoic acid	Cayman Chemical	38600	9.1	295.2 > 195.0	d4-13-HODE
13-HOTrE	13(S)-HOTrE	13S-hydroxy-9Z,11E,15Z-octadecatrienoic acid	Cayman Chemical	39620	8.1	293.2 > 195.2	d4-13-HODE
13-HOTrEγ	13(S)-HOTrEγa	13S-hydroxy-6Z,9Z,11E-octadecatrienoic acid	Cayman Chemical	39610	8.3	293.2 > 193.0	d4-13-HODE
13-KODE	13-oxo-ODE	13-oxo-9Z,11E-octadecadienoic acid	Cayman Chemical	38620	9.5	293.2 > 112.9	d3-9-KODE
14(15)-EpETrE	(±)14,15-EpETrE	(±)14,15-epoxy-5Z,8Z,11Z-eicosatrienoic acid	Cayman Chemical	50651	10.8	319.3 > 219.1	d11-11(12)-EpETrE
14,15-DiHETE	(±)14,15-DiHETE	(±)14,15-dihydroxy-5Z,8Z,11Z,17Z-eicosatetraenoic acid	Cayman Chemical	10006998	6.3	335.2 > 207.1	d11-11,12-DiHETrE
14,15-DiHETrE	(±)14,15-DiHETrE	(±)14,15-dihydroxy-5Z,8Z,11Z-eicosatrienoic acid	Cayman Chemical	51651	7.2	337.3 > 207.0	d11-14,15-DiHETrE
14-HDoHE	(±)14-HDoHE	(±)14-hydroxy-4Z,7Z,10Z,12E,16Z,19Z-docosahexaenoic acid	Cayman Chemical	33550	9.8	343.2 > 281.2	d8-15-HETE
15-HEDE	(±)15-HEDE	(±)15-hydroxy-11Z,13E-eicosadienoic acid	Cayman Chemical	37700	11.1	323.2 > 223.1	d4-13-HODE
15-HEPE	(±)15-HEPE	(±)15-hydroxy-5Z,8Z,11Z,13E,17Z-eicosapentaenoic acid	Cayman Chemical	32700	8.5	317.1 > 219.1	d8-15-HETE
15-HETE	(±)15-HETE	(±)15-hydroxy-5Z,8Z,11Z,13E-eicosatetraenoic acid	Cayman Chemical	34700	9.3	319.1 > 174.8	d8-15-HETE
15-HETrE	15(S)-HETrEa	15S-hydroxy-8Z,11Z,13E-eicosatrienoic acid	Cayman Chemical	36720	10.1	321.3 > 221.1	d8-15-HETE
15-KETE	15-oxo-ETE	15-oxo-5Z,8Z,11Z,13E-eicosatetraenoic acid	Cayman Chemical	34730	9.7	317.2 > 112.9	d6-5-KETE
16(17)-EpDPE	(±)16,17-EpDPE	(±)16,17-epoxy-4Z,7Z,10Z,13Z,19Z-docosapentaenoic acid	Cayman Chemical	10174	10.9	343.3 > 233.2	d11-11(12)-EpETrE
17,18-DiHETE	(±)17,18-DiHETE	(±)17,18-dihydroxy-5Z,8Z,11Z,14Z-eicosatetraenoic acid	Cayman Chemical	10006999	5.9	335.2 > 247.1	d11-14,15-DiHETrE
17-HDoHE	(±)17-HDoHE	(±)17-hydroxy-4Z,7Z,10Z,13Z,15E,19Z-docosahexaenoic acid	Cayman Chemical	33650	9.5	343.2 > 281.2	d8-15-HETE
18-HEPE	(±)18-HEPE	(±)18-hydroxy-5Z,8Z,11Z,14Z,16E-eicosapentaenoic acid	Cayman Chemical	32840	8.1	317.2 > 215.1	d8-15-HETE
19(20)-EpDPE	(±)19,20-EpDPE	(±)19,20-epoxy-4Z,7Z,10Z,13Z,16Z-docosapentaenoic acid	Cayman Chemical	10175	10.5	343.3 > 281.2	d11-11(12)-EpETrE
19,20-DiHDPA	(±)19,20-DiHDPA	(±)19,20-dihydroxy-4Z,7Z,10Z,13Z,16Z-docosapentaenoic acid	Cayman Chemical	10007001	7.2	361.2 > 229.1	d11-14,15-DiHETrE
4-HDoHE	(±)4-HDoHE	(±)4-hydroxy-5E,7Z,10Z,13Z,16Z,19Z-docosahexaenoic acid	Cayman Chemical	33200	10.7	343.2 > 101.0	d8-5-HETE
5,6-DiHETrE	(±)5,6-DiHETrE	(±)5,6-dihydroxy-8Z,11Z,14Z-eicosatrienoic acid	Cayman Chemical	51211	8.7	337.3 > 144.9	d11-8,9-DiHETrE
5-HEPE	(±)5-HEPE	(±)5-hydroxy-6E,8Z,11Z,14Z,17Z-eicosapentaenoic acid	Cayman Chemical	32200	9.1	317.3 > 114.8	d8-5-HETE
5-HETE	(±)5-HETE	(±)5-hydroxy-6E,8Z,11Z,14Z-eicosatetraenoic acid	Cayman Chemical	34210	10.5	319.1 > 114.9	d8-5-HETE
5-HETrE	5(S)-HETrEa	5S-hydroxy-6E,8Z,11Z-eicosatrienoic acid	Cayman Chemical	36230	11.9	321.3 > 205.1	d8-5-HETE
5-iPF2α-VI	(±)5-iPF2α-VI	5,9α,11α-trihydroxy-(8β)-prosta-6E,14Z-dien-1-oic acid	Cayman Chemical	16300	2.3	353.2 > 114.9	d11-5-iPF2a-VI
5-KETE	5-oxo-ETE	5-oxo-6E,8Z,11Z,14Z-eicosatetraenoic acid	Cayman Chemical	34250	11.0	317.2 > 203.1	d6-5-KETE
7,17-hydroxy-DPA	7(S),17(S)-hydroxy DPAa	7,17-dihydroxy-8E,10Z,13Z,15E,19Z-docosapentaenoic acid	Cayman Chemical	37730	6.4	361.2 > 143.0	d11-14,15-DiHETrE
8(9)-EpETrE	(±)8,9-EpETrE	(±)8,9-epoxy-5Z,11Z,14Z-eicosatrienoic acid	Cayman Chemical	50351	11.3	319.3 > 155.0	d11-8(9)-EpETrE
8,9-DiHETrE	(±)8,9-DiHETrE	(±)8,9-dihydroxy-5Z,11Z,14Z-eicosatrienoic acid	Cayman Chemical	51351	8.1	337.2 > 126.9	d11-8,9-DiHETrE
8-HDoHE	(±)8-HDoHE	(±)8-hydroxy-4Z,6E,10Z,13Z,16Z,19Z-docosahexaenoic acid	Cayman Chemical	33350	10.2	343.2 > 189.0	d8-5-HETE
8-HEPE	(±)8-HEPE	(±)8-hydroxy-5Z,9E,11Z,14Z,17Z-eicosapentaenoic acid	Cayman Chemical	32340	8.7	317.0 > 154.9	d8-12-HETE
8-HETE	(±)8-HETE	(±)8-hydroxy-5Z,9E,11Z,14Z-eicosatetraenoic acid	Cayman Chemical	34340	10.0	319.0 > 154.9	d8-12-HETE
8-HETrE	8(S)-HETrEa	8S-hydroxy-9E,11Z,14Z-eicosatrienoic acid	Cayman Chemical	36360	10.5	321.3 > 157.0	d8-5-HETE
9,10-DiHOME (erythro)	(±)9,10-DiHOME	(±)9,10-dihydroxy-12Z-octadecenoic acid	Cayman Chemical	53400	6.5	313.2 > 201.1	d4-9,10-DiHOME

9,10-DiHOME (threo)	(±)9,10-DiHOME	(±)9,10-dihydroxy-12Z-octadecenoic acid	Cayman Chemical	53400	6.9	313.2 > 201.1	d4-9,10-DiHOME
9-HETE	(±)9-HETE	(±)9-hydroxy-5Z,7E,11Z,14Z-eicosatetraenoic acid	Cayman Chemical	34400	10.2	319.2 > 179.1	d8-12-HETE
9-HODE	(±)9-HODE	(±)9-hydroxy-10E,12Z-octadecadienoic acid	Cayman Chemical	38400	9.2	295.3 > 171.0	d4-9-HODE
9-HOTrE	9(S)-HOTrEa	9S-hydroxy-10E,12Z,15Z-octadecatrienoic acid	Cayman Chemical	18500	7.9	293.2 > 170.9	d4-9-HODE
9-KODE	9-KODE	9-oxo-10E,12Z-octadecadienoic acid	Cayman Chemical	38420	9.7	293.2 > 185.0	d3-9-KODE
9-KOTrE	9-KOTrE	9-oxo-10E,12Z,15Z-octadecatrienoic acid	Cayman Chemical	10546	8.6	291.2 > 185	d3-9-KODE
12(13)-EpOME (cis)	(±)12,13-EpOME	(±)12,13-epoxy-9Z-octadecenoic acid	Cayman Chemical	52450	10.6	295.2 > 194.9	d4-9(10)-EpOME
9(10)-EpOME (cis)	(±)9,10-EpOME	(±)9,10-epoxy-12Z-octadecenoic acid	Cayman Chemical	52400	10.8	295.2 > 170.9	d4-9(10)-EpOME
LXA4 / epiLXA4	LXA4	5S,6R,15S-trihydroxy-7E,9E,11Z,13E-eicosatetraenoic acid	Cayman Chemical	20410	3.7	351.2 > 115	d5-LXA4
PGD2	PGD2	9α,15S-dihydroxy-11-oxo-prosta-5Z,13E-dien-1-oic acid	Cayman Chemical	12010	3.0	351.2 > 271.1	d4-PGD2
PGE1	PGE1	9-oxo-11α,15S-dihydroxy-prost-13E-en-1-oic acid	Cayman Chemical	13010	2.8	353.3 > 317.3	d4-PGD1
PGE2	PGE2	9-oxo-11α,15S-dihydroxy-prosta-5Z,13E-dien-1-oic acid	Cayman Chemical	14010	2.7	351.2 > 271.1	d4-PGE2
PGF2α	PGF2α	9α,11α,15S-trihydroxy-prosta-5Z,13E-dien-1-oic acid	Cayman Chemical	16010	2.5	353.3 > 193.0	d4-8-epi-PGF2a
TXB2	TXB2	9α,11,15S-trihydroxythromba-5Z,13E-dien-1-oic acid	Cayman Chemical	19030	2.0	369.2 > 168.9	d4-TBX2
TXB3	TXB3	9α,11,15S-trihydroxy-thromba-5Z,13E,17Z-trien-1-oic acid	Cayman Chemical	19990	1.6	367.2 > 195.15	d4-TBX2
**Screened but not reported**							
**Compound Name**	**Standard**	**Systematic Name**	**Supplier**	**Cat. Number**	**Retention time (min.)**	**SRM transition**	**Internal Standard**
19-HETE	19(S)-HETE	(5Z,8Z,11Z,14Z)-19-hydroxy-5,8,11,14-icosatetraenoic acid	Cayman Chemical	10007766	8.23	319.2 > 275.1	d6-20-HETE
20-HETE	20-HETE	20-hydroxy-5Z,8Z,11Z,14Z-eicosatetraenoic acid	Cayman Chemical	90030	8.42	319.2 > 275.1	d6-20-HETE
8-iso-PGE2	8-iso-PGE2	9-oxo-11α,15S-dihydroxy-(8β)-prosta-5Z,13E-dien-1-oic acid	Cayman Chemical	14350	2.6	351.2 > 271.1	d4-isoPGE2
9,10,13-TriHOME	9(S),10(S),13(S)-TriHOMEa	9S,10S,13S-trihydroxy-11E-octadecenoic acid	Larodan	14-1802-15	2.5	329.0 > 139.0	d4-PGE2
9,12,13-TriHOME	9(S),12(S),13(S)-TriHOME	9S,12S,13S-trihydroxy-10E-octadecenoic acid	Larodan	14-1802-14	2.4	329.1 > 211.0	d4-PGE2
LTE4	LTE4	5S-hydroxy-6R-(S-cysteinyl)-7E,9E,11Z,14Z-eicosatetraenoic acid	Cayman Chemical	20410	5.37	438.4 > 333.3	d5-LTE4
LXA5	LXA5	5S,6R,15S-trihydroxy-7E,9E,11Z,13E,17Z-eicosapentaenoic acid	Cayman Chemical	90410	2.7	349.2 > 114.9	d5-LXA4
PGD1	PGD1	9α,15S-dihydroxy-11-oxo-prost-13E-en-1-oic acid	Cayman Chemical	12000	2.94	353.3 > 317.3	d4-PGD2
PGD3	PGD3	9α,15S-dihydroxy-11-oxo-prosta-5Z,13E,17Z-trien-1-oic acid	Cayman Chemical	20110	2.22	349.3 > 269.3	d4-PGD1
PGE3	PGE3	9-oxo-11α,15S-dihydroxy-prosta-5Z,13E,17Z-trien-1-oic acid	Cayman Chemical	14990	2.04	349.3 > 269.3	d4-PGE2
12(13)-EpOME (trans)	(±)12,13-EpOME	(±)12,13-epoxy-9Z-octadecenoic acid	Cayman Chemical	52450	10.8	295.2 > 194.9	d4-9(10)-EpOME
9(10)-EpOME (trans)	(±)9,10-EpOME	(±)9,10-epoxy-12Z-octadecenoic acid	Cayman Chemical	52400	11	295.2 > 170.9	d4-9(10)-EpOME

ESM Table 2. General and chromatographic method details for the analysis of reported oxylipins. SRM: selected reaction monitoring.

Species	Tissue	Reference	Platform	Description
Human	Skeletal muscle	GSE59363	Affymetrix Human Gene 1.0 ST Array	Men with or without type 2 diabetes before exercise, immediately after exercise and 3h after an acute bout of exercise.
		GSE202295	Illumina HiSeq 2500	Men with or without type 2 diabetes before exercise, immediately after exercise and 3h after an acute bout of exercise.
		GSE214544	NextSeq 2000	Single cell RNAseq of skeletal muscle from healthy men before and 3h after an acute bout of exercise.
		MetaMEx	Various	Meta-analysis of skeletal muscle response to exercise, including male and female volunteers.
	Whole blood	GSE51216	Agilent-014850 Whole Human Genome Microarray 4x44K	Blood samples from control and athlete men were analysed before exercise, immediately after exercise and 24 hours after exercise.
		GSE164890	Illumina HiSeq 4000	Blood samples were collected from young elite speed skater men, before and after exercise.
	Peripheral blood mononuclear cells	GSE3606	Affymetrix Human Genome U133A 2.0 Array	Blood samples were collected from healthy male probands before and one hour past an acute exercise bout (treadmill, 60% VO2max).
		GSE6053	Affymetrix Human Genome U133A 2.0 Array	Blood samples were collected from healthy male probands before and one hour after an exhaustive treadmill test (80% VO2max).
		GSE101931	Illumina HumanHT-12 V4.0 expression beadchip	Blood samples were collected from healthy individuals before exercise and 1h, 6h, and 24h after exercise
Mouse	BAT, Heart, WAT, Liver, Muscle	GSE92719	Rosetta/Merck Mouse 38.4K RSTA Affymetrix	C57BL/6 male mice, vastus lateralis, heart, liver, brown and white adipose tissues collected immediately after treadmill running.
	Liver	GSE110747	Affymetrix Mouse Gene 2.1 ST	C57BL/6N male mice, liver collected immediately after treadmill exercise.
	Muscle	GSE117161	Illumina NextSeq 500	C57B6/J male mice, gastrocnemius muscle collected immediately after treadmill exercise
		GSE126962	Illumina HiSeq 2500	C57B6/J male mice, gastrocnemius muscle collected immediately after treadmill exercise
		GSE178262	Illumina HiSeq 500	C57BL/6 male mice, quadriceps muscle collected immediately after treadmill exercise.

ESM Table 3. Publicly available datasets included in the analysis

Figures

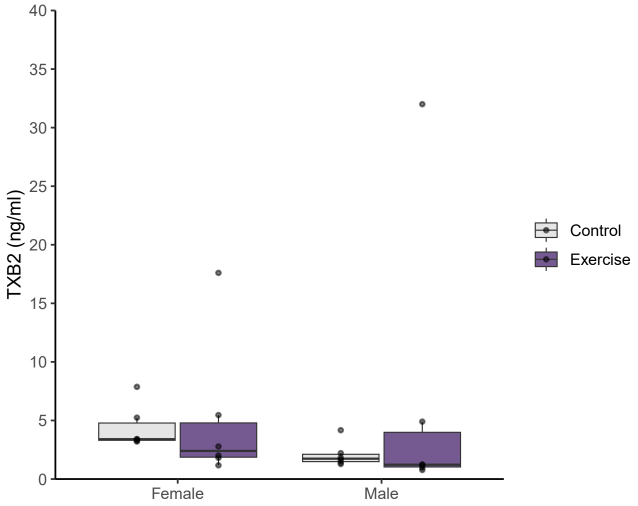

ESM Figure 1. Thromboxane levels in plasma from male and female mice before and after an acute bout of exercise. Plasma was collected from female and male mice before and after an acute bout of treadmill exercise and thromboxane B2 (TXB2) measured using mass spectrometry as described in supplementary methods. Results are box and whisker plots, n = 6. No significant comparisons were found using two-way ANOVA (sex, exercise).

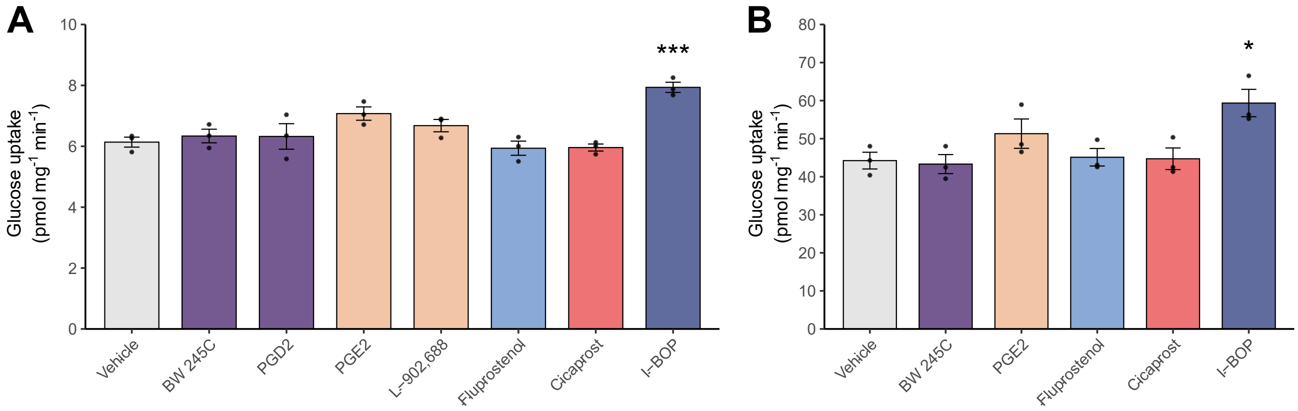

ESM Figure 2. Glucose uptake in rat L6 myotubes and mouse C2C12 in response to prostanoid receptor agonists. Glucose uptake was measured using radiolabelled 2-deoxy-glucose as described in methods. A. L6 myotubes, n = 3. B. Mouse C2C12 myotubes, n = 3. Results are mean, SEM and individual data points. One-way ANOVA with Tukey's HSD test comparing each agonist to the vehicle, *, p<0.05, ***p<0.001.

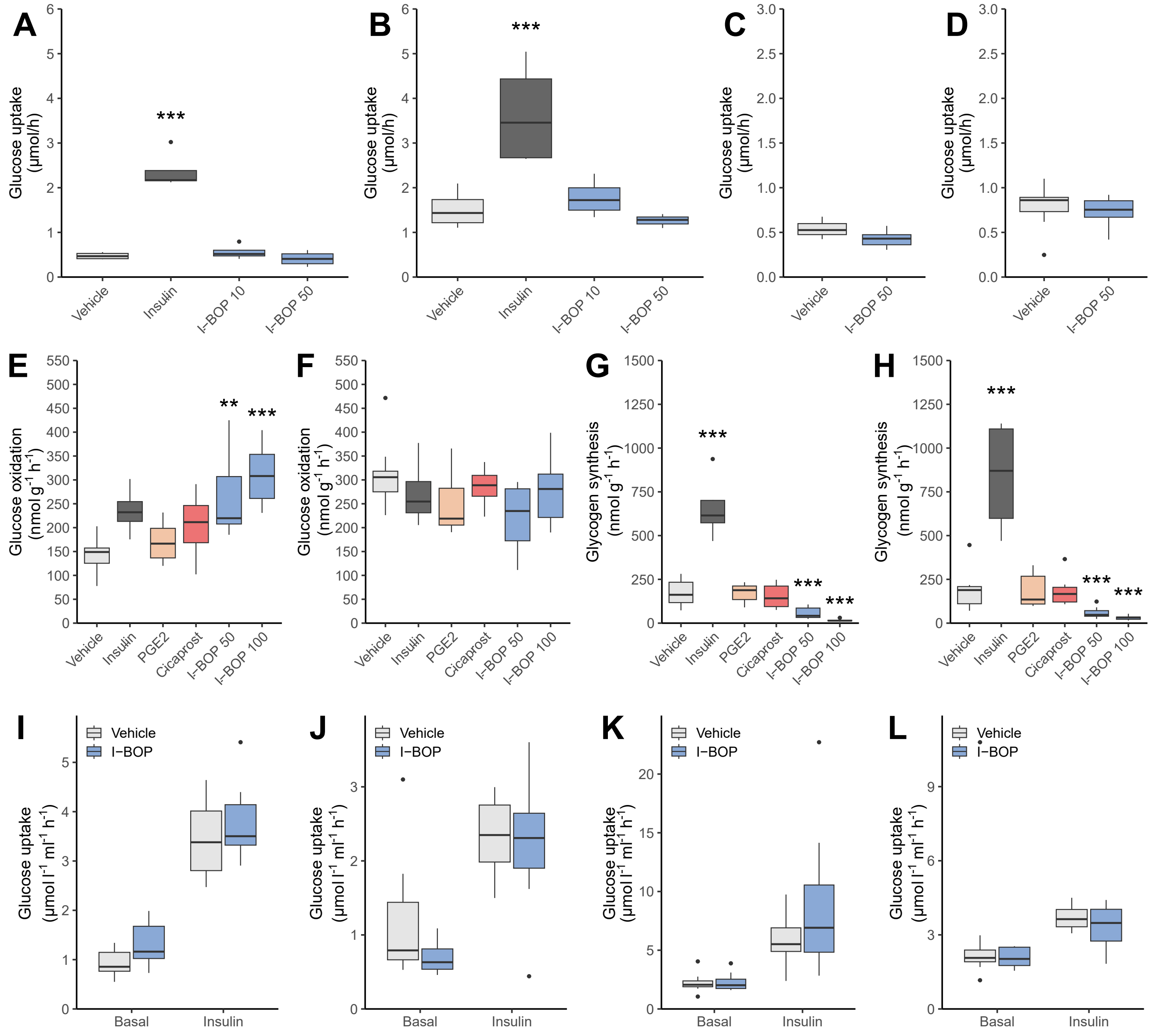

ESM Figure 3. Thromboxane receptor stimulation ex vivo does not affect glucose uptake in mouse Soleus and EDL muscles. A-B. EDL (A) and soleus (B) mouse muscles were isolated and incubated with 10 µM I-BOP (I-BOP-10) or 50 µM (I-BOP-50) *ex vivo* for 30 minutes. Glucose uptake was measured with radiolabelled 2-deoxy-glucose. Results are box and whisker plots, n = 4. Kruskal-Wallis with Tukey's HSD test comparing each agonist to the vehicle, ***p<0.001. **C-D.** EDL (C) and soleus (D) mouse muscles were isolated and incubated with 50 µM I-BOP *ex vivo* for 3 hours. Glucose uptake was measured with radiolabelled 2-deoxy-glucose. Data are presented as box-and-whisker plots with individual data points and were analysed using the Wilcoxon signed-rank test, n = 7. **E-F.** EDL (E) and Soleus (F) mouse muscles were isolated and incubated *ex vivo* with 50 µM prostanoid agonists or 100 µM I-BOP (I-BOP-100) for 3 hours. Glucose uptake and oxidation was measured with radiolabelled glucose. Results are box and whisker plots, n =8 for vehicle and agonists, n=4 for insulin. Kruskal-Wallis with Tukey's HSD test comparing each agonist to the vehicle, **p<0.01, ***p<0.001. **G-H.** EDL (G) and soleus (H) mouse muscles were isolated and incubated *ex vivo* with 50 µM prostanoid agonists or 100 µM I-BOP (I-BOP-100) for 3 hours. Glucose incorporation into glycogen was measured with radiolabelled glucose. Results are box and whisker plots, n =8 for vehicle and agonists, n=4 for insulin. Kruskal-Wallis with Tukey's HSD test comparing each agonist to the vehicle, ***p<0.001. **I-L.** Female (I,K) and male (J-L) mice were injected with 20 µg/kg I-BOP. After 1h, EDL (I-J) and soleus (K-L) muscles were collected and incubated *ex vivo* to measure glucose uptake with radiolabelled 2-deoxyglucose at baseline and in response to insulin. Results are box and whisker plots, n = 8, 3-way ANOVA (I-BOP, insulin, sex). The only significant post-hoc comparisons were Insulin compared to Basal.

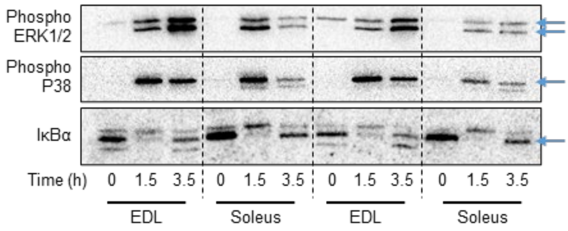

ESM Figure 4. Activation of stress and inflammatory signalling pathways in isolated mouse skeletal muscle incubated ex vivo. Extensor digitorum longus (EDL) and soleus muscles were either snap frozen after dissection (0 h) or incubated *ex vivo* for 1.5 h or 3.5 h. Western blot analysis was performed on muscle lysates to assess stress and inflammatory signalling pathways. Representative blots are shown for phospho-ERK1/2 (Thr202/Tyr204), phospho-p38 (Thr180/Tyr182) and IκBα (total). Data are representative of n = 2 mice per group.

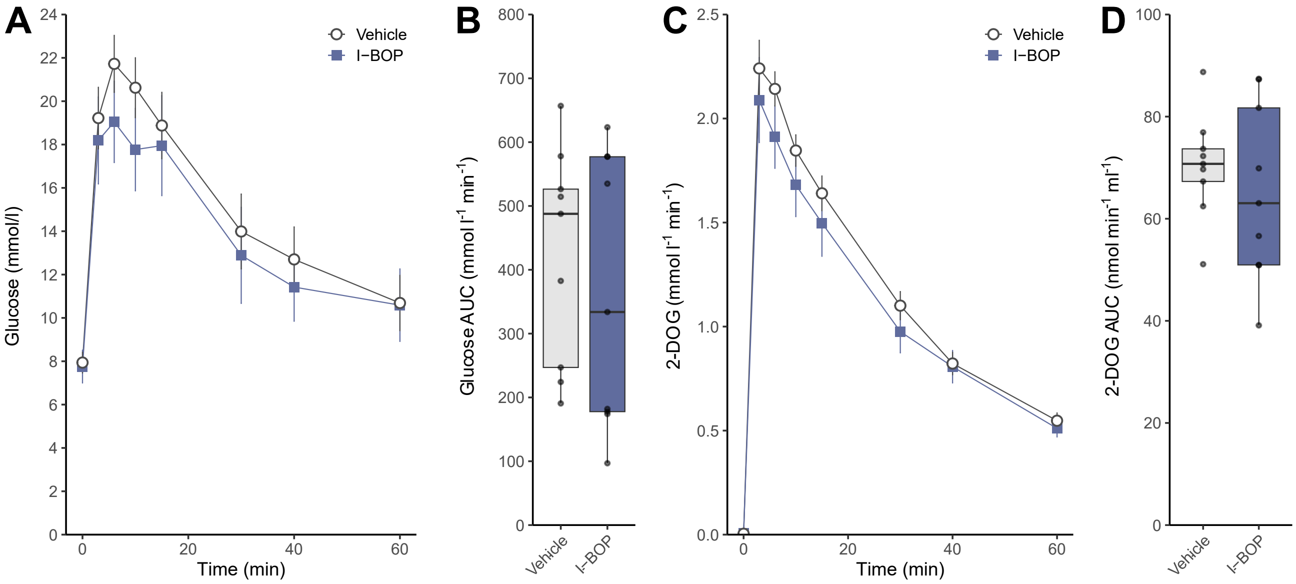

ESM Figure 5. Thromboxane stimulation *in vivo*. A. Blood glucose concentration after bolus injection. **B.** Area under the curve of blood glucose presented in panel A. **C.** Concentration of ^{14}C-2-deoxyglucose (2-DOG) in the blood after bolus injection. **D.** Area under the curve of ^{14}C-2-deoxyglucose presented in panel C. Results are mean +/- SEM, n = 8-9.
